# Dimensional cophenetic integrity: a method for evaluation of dimensionality reduction in MSI

**DOI:** 10.1093/bioadv/vbag100

**Published:** 2026-04-15

**Authors:** Connor J Newstead, Josephine Bunch, Melanie J Bailey, Alex Dexter

**Affiliations:** National Centre of Excellence in Mass Spectrometry Imaging (NiCE-MSI), National Physical Laboratory (NPL), Teddington, TW11 0LW, United Kingdom; School of Chemistry & Chemical Engineering, University of Surrey, Guildford, GU2 7XH, United Kingdom; National Centre of Excellence in Mass Spectrometry Imaging (NiCE-MSI), National Physical Laboratory (NPL), Teddington, TW11 0LW, United Kingdom; Faculty of Medicine, Department of Metabolism, Digestion and Reproduction, Imperial College London, London, SW7 2AZ, United Kingdom; Department of Infectious Diseases, Faculty of Life Science and Medicine, Kings College London, London, WC2R 2LS, United Kingdom; Department of Molecular and Systems Biology, Faculty of Health and medical sciences, University of Surrey, Guildford, GU2 7XH, United Kingdom; National Centre of Excellence in Mass Spectrometry Imaging (NiCE-MSI), National Physical Laboratory (NPL), Teddington, TW11 0LW, United Kingdom

## Abstract

**Motivation:**

Mass spectrometry imaging data typically contains tens of thousands of pixels, and m/z channels which may relate to biomolecules of interest. It is impossible to visualize such highly dimensional data, and many multi-variate analyses cannot be conducted without reducing dimensionality. dimensionality reduction algorithms are commonly used for data visualisation, feature selection and as part of data clustering workflows in examination of large Mass spectrometry imaging datasets. In this work, we seek to develop methods to determine the ability of dimensionality reduction algorithms to preserve local and global structure within reduced data.

**Results:**

We have developed a novel evaluation method—Dimensional Cophenetic Integrity which measures the structure and pattern preservation of dimensionality reduction algorithms based on cophenetic distance of hierarchically clustered samples. We demonstrate that Dimensional Cophenetic Integrity results are indicative of expected tissue segmentation and image quality when compared to known synthetic data. Additionally, we find that optimum dimensionality reduction embeddings derive from hyperparameter selection far outside the typical range and show that Dimensional Cophenetic Integrity can be used as an objective criterion for Bayesian optimization. It is shown that optimization of dimensionality reduction preserve cluster relationships compared to default dimensionality reduction algorithm parameter decisions.

## 1 Introduction

Mass spectrometry imaging (MSI) is a powerful collection of techniques to probe the molecular composition of a surface in a spatially resolved manner (Caprioli *et al.* 1997). It can be used to measure a wide range of molecular classes including drugs, metabolites, lipids, and proteins (Eberlin *et al.* 2011, Dexter *et al.* 2019) to derive better understanding of diseases and treatments, as well as to discover new biomarkers and drug targets (Najumudeen *et al.* 2021, Vande Voorde *et al.* 2023). One of the primary challenges in MSI is the vast amount of information that is available. A typical MSI experiment can contain hundreds of thousands of pixels, and tens of thousands of m/z channels, each of which may relate to a possible molecule of interest i.e. a potential biomarker. This is comparable to the size and complexity of other omics data such as spatial transcriptomics, and proteomics, as well as single cell trancriptomics, proteomics and metabolomics. This means that developments in methods applied to MSI are readily transferrable to other omics studies (Dexter *et al.* 2025). One of the common methods to assist in mining and interpreting these vast amounts of data are dimensionality reduction algorithms such as principal component analysis (PCA) (McCombie *et al.* 2005). Dimensionality reduction in MSI seeks to summarise this vast quantity of information by reduction of feature space for visualisation in 2 or 3 dimensions and can be used to rapidly stratify phenotypic differences (Abdelmoula *et al.* 2016).

The quantity of data in MSI continues to increase, due to the introduction of higher spatial and spectral resolution mass spectrometry. Therefore, strategies to efficiently process and interpret such volumes of high dimensional data are vital (Römpp and Spengler 2013).

It is impossible to visualise thousands of m/z channel intensities across thousands of pixels in an imaging experiment to identify patterns or biological regions of interest. In the field of MSI, the phenomenon known as ‘the curse of dimensionality’ can refer to problems faced by sparsity of data, computational complexity, problems with visualisation amongst many others (Bellman 1966).

t-distributed stochastic neighbour embedding (t-SNE) is the most well-known and popular dimensionality reduction algorithm in MSI and many other fields (Abdelmoula *et al.* 2016, Balluff *et al.* 2022, Prasad *et al.* 2022, Dexter *et al.* 2025). The use of non-linear dimensionality reduction methods such as t-SNE and other more recent methods such as uniform manifold approximation projection (McInnes *et al.* 2020) (UMAP) has become increasingly popular due to their superior ability to identify trends in the data not discovered using linear methods such as PCA (Abdelmoula *et al.* 2016, Smets *et al.* 2019). Other non-linear dimensionality reduction methods have been introduced for MSI such as autoencoders, and other neural network based algorithms however neural network based approaches are not discussed within this work because these methods learn a parametric function which depend on the neural network architecture, training, and learned weight. T-SNE and UMAP have fixed mathematical objectives that do not learn a parametric function to map input data to outputs, and are therefore more generalizable approaches to demonstrate our evaluation method. Whilst neural networks and autoencoders are less common in MSI and not discussed within this work, we acknowledge that our evaluation method should still be applicable to these methods. Whilst kernel-based methods such as support vector machines (SVM) have been used for classification in MSI (Galli *et al.* 2016, Meding *et al.* 2012) these require some form of labelling, and to date, there are no documented uses of other kernel based dimensionality reduction such as kernel PCA (k-PCA) in MSI potentially due to the fact that k-PCA has low interpretability (Liebal *et al.* 2020, Briscik *et al.* 2023). Other approaches also consider the spatial information of high dimensional imaging data such as maximum autocorrelation factor (MAF) (Henderson *et al.* 2009) and spatially aware clustering (Alexandrov and Kobarg 2011) but these approaches are less common than methods that do not consider the additional spatial domain. This is likely due to the additional computational complexity associated with the incorporation of the additional spatial information. As with methods that do not consider the additional spatial information, a robust evaluation metric to objectively assess the performance of these methods is critical.

Two of the most common uses of t-SNE and UMAP in MSI is for use in image registration and as a precursor to clustering. Image registration between two datasets is often used to perform alignment of two images such as MSI to histopathology. In image registration landmark features can be selected which should represent the same physical location for each dataset. An image of a single variable, in this case a peak at one specific m/z is often selected, but a reduced representation using a dimensionality reduction algorithm produces a more complete representation of features that are much easier to match between modalities (Abdelmoula *et al.* 2019). Clustering of MSI datasets is often performed to distinguish between areas of biological similarity, or anatomical regions. As with registration, the use of dimensionality reduction prior to clustering improves the resulting segmentation and can be used to study tumour sub-populations for example (Abdelmoula *et al.* 2016).

Considering that DR algorithms are used as such an important pre-processing step in the analysis of MSI data, it is crucial that the embedding (representation of data in lower dimensional space) preserves the structure and patterns within the data. Loss of information, or lack of preservation of patterns and structure in the data could lead to misalignment in registration, or clustering which does not represent all regions of variability or misrepresents the relationships between clusters. Having objective measures of embedding performance can also be used for appropriate selection of tuneable algorithm parameters (hyperparameters) and can be used to evaluate new dimensionality reduction algorithms as they arise.

### 1.1 Methods to evaluate dimensionality reduction

When performing dimensionality reduction there is an inherent trade off in the preservation of localised features (retaining neighbourhood of similar spectra) and global features (relative separation of clusters) with different algorithms performing better or worse at these different aspects. For example, t-SNE often performs well at the local feature scale, while UMAP outperforms t-SNE on global feature preservation (Wang *et al.* 2021). It is also important to note the values used for hyperparameters can favour local Versus global feature preservation (Gove *et al.* 2022). The most common example of this is the perplexity used in t-SNE which will greatly influence the preservation of global or local features (Cao and Wang 2017).

The evaluation of quality of dimensionality reduction algorithms is an important field of research with many different evaluation methods. In the current use of dimensionality reduction in MSI, the biological outcome or image autocorrelation have been used as the means to evaluate the performance when performing t-SNE and UMAP respectively (Abdelmoula *et al.* 2016, Smets *et al.* 2019). Brunet *et al.* evaluate the robustness of clustering results obtained after using nonnegative matrix factorization (NMF) by using cophenetic correlation (the correlation of the distances between data points and their respective distances in a dendrogram) between consensus matrix sample distances, and the sample distances from the linkage in the reordering of the consensus matrix (Brunet *et al.* 2004). This study demonstrated that the cophenetic distance is a useful metric in the measurement of sample-cluster relationships. Cophenetic distance is a metric which measures the similarity between two data points based on their height (distance) at which they become part of the same cluster in a hierarchical clustering dendrogram (Sokal and Rohlf 1962). The quality of DR algorithms is commonly evaluated by visual inspection of expected data separation. An example of this is shown by Popov et al (Zhvansky *et al.* 2021).where the greatest DR algorithm was deemed to be that which lead to the greatest contrast in organized spectra similarity matrices (Zhvansky *et al.* 2021, 2019). The common problem with evaluations such as this is that the data sample relationships are not measured, and the preservation of such relationships is the fundamental goal of a DR algorithm which our method aims to address.

Other methods for the evaluation of DR algorithms have been developed in other fields, however many require labelled datasets and are therefore unable to be used for many biological studies, including those in MSI. Two examples of methods that use labelled data are the Centroid Distance Correlation (CDC) which is the Spearman correlation between high and low dimensional cluster centroid pairwise distances (Kobak and Berens 2019, Huang *et al.* 2022), and the mean-based Dunn Index which calculates the ratio between mean inter cluster distances and mean intra cluster distances (Dunn 1973). A good embedding will show clear separation of the regions, and thus the inter region distance should be higher than the intra region distance, thereby giving a mean Dunn index >1. For methods such as the CDC prior labels could be generated using clustering, however this introduces further uncertainty in which clustering algorithm is most appropriate, and how to determine the correct number of clusters in the dataset, neither of which are comprehensively evaluated in MSI.

For unlabelled datasets, which are most common in biological studies unsupervised methods are required, these include methods such as the three described below. Full details of these methods in addition to others are provided in the Table 1, available as supplementary data at *Bioinformatics Advances* online.

- Global feature preservation can be evaluated using random triplet (RT) accuracy, which assesses if the relative order of triplets of pixels are the same (Wang *et al.* 2021).
- Local feature comparison can be made by comparing the co-k-nearest neighbours (KNN), which measures the number of co-occurring nearest neighbours for every pixel (Huang *et al.* 2022).
- Global feature preservation can also be evaluated by calculating the Spearman rank between high and low dimensional pairwise distances.

In MSI, often arbitrary or default hyperparameters (examples in Table 1, available as supplementary data at *Bioinformatics Advances* online) are selected without an objective optimisation criterion. One alternative way to compare these algorithms is against the overall goal of the methods themselves. In dimensionality reduction this means that the low dimensional embedding retains the characteristics of its high dimensional counterpart. Here, we introduce Dimensional Cophenetic Integrity, a new evaluation method which aims to evaluate the preservation of cluster relationships in MSI. We describe the use of this method on a selection of previously published real and synthetic MSI datasets and have selected the two most popular algorithms, t-SNE and UMAP (Dexter *et al.* 2016, McInnes *et al.* 2020, Race *et al.* 2020, Vande Voorde *et al.* 2023). This sets out the framework to perform objective comparisons of different dimensionality reduction algorithms in the field of MSI, as well as the potential to automate the optimisation of aspects such as hyperparameters.

## 2 Methods

### 2.1 Synthetic data

#### 2.1.1 Synthetic matrix assisted laser desorption ionisation (MALDI) data

**Figure 1:**
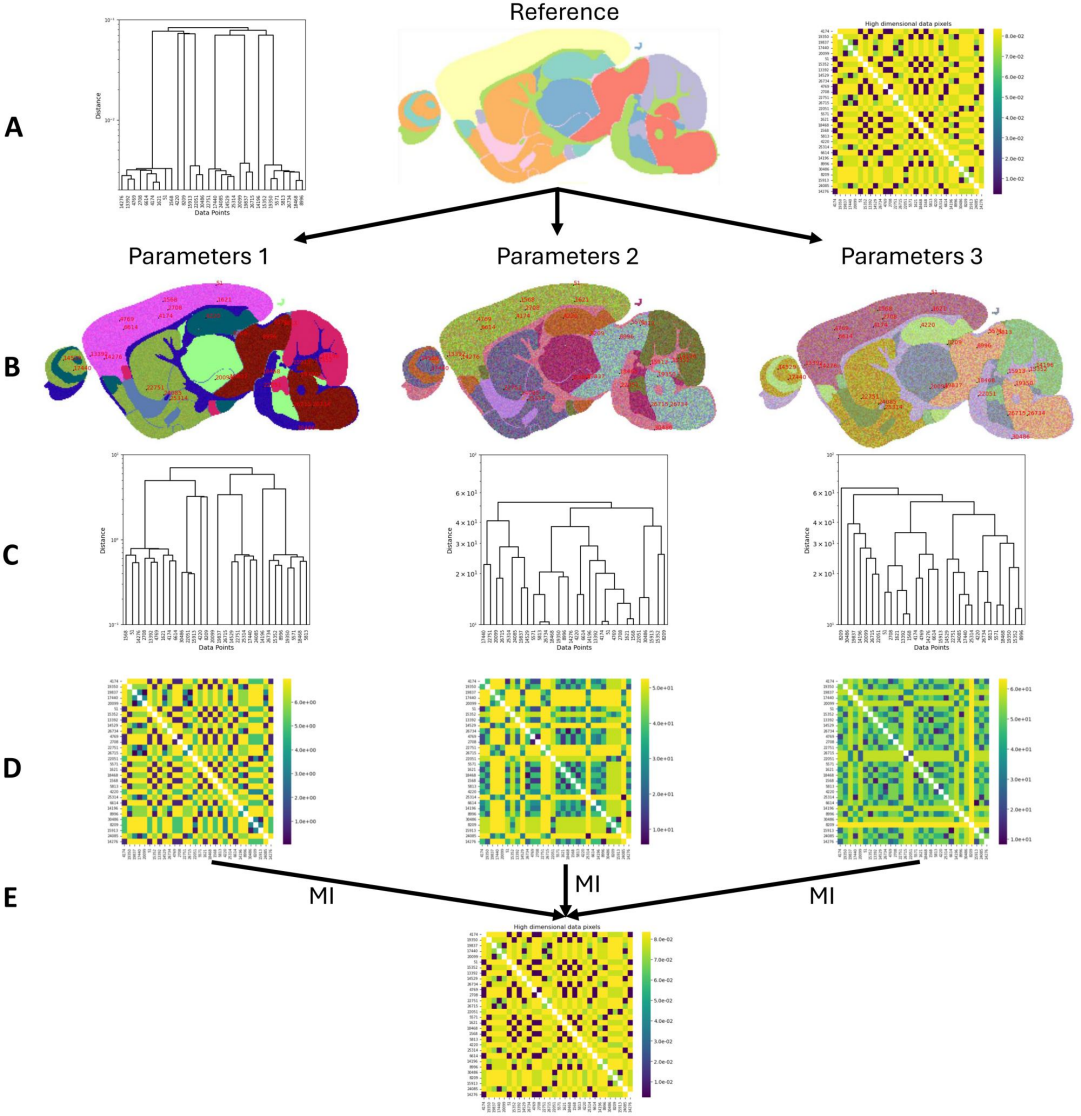
(A) Original data (high dimensional) samples hierarchical clustering dendrogram, known 3D reduced representation reference image, high dimensional pairwise cophenetic distance matrix between samples (left to right respectively). (B) t-SNE embeddings of the synthetic MALDI dataset. (C) Reduced representation embedding (low dimensional) samples hierarchical clustering dendrograms of their associated embedding (above). (D) Low dimensional pairwise cophenetic distance matrix between samples. (E) Mutual information between the low dimensional cophenetic distance matrix and high dimensional cophenetic distance matrix.

**Figure 2:**
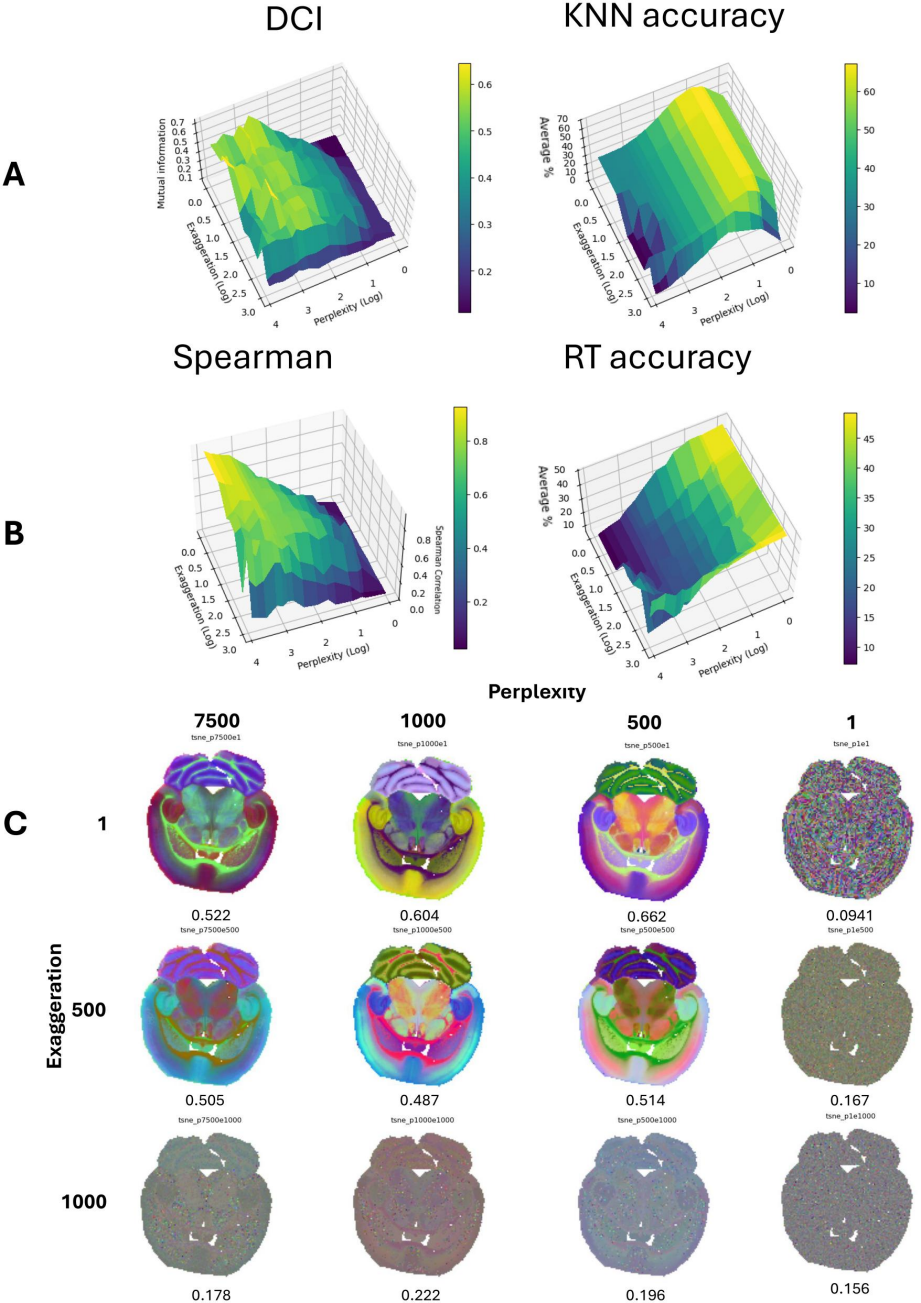
(A) Gridplot of DCI, and KNN accuracy for a range of perplexity and exaggeration results, respectively. (B) Gridplot of Spearman Correlation, and RT Accuracy for a range of perplexity and exaggeration results, respectively. (C) 3D reduced representation embeddings of a range of Perplexity and Exaggeration with their associated DCI results.

The synthetic MALDI data were created using a previously described approach (Race *et al.* 2020). In brief, metabolites were chosen from selected biochemical pathways (see supplementary material excel file) from the human metabolome database (Wishart *et al.* 2022) (HMDB) along with possible adducts of [M + H]+, [M+Na]+ and [M + K]+(Wishart *et al.* 2022). Theoretical isotope distributions were then created for each of the metabolite and adduct combinations using the Matlab ‘isotopicdist’ function (MATLAB 2017a and Bioinformatics Toolbox, The MathWorks), and mass spectra were generated using the peaks associated with these different mass to charge values. An exemplar total mass spectrum from these data is provided in Fig. 1, available as supplementary data at *Bioinformatics Advances* online. A region mask was generated using a labelled sagittal section image from the Allen Mouse Brain Atlas (Jones *et al.* 2009). Different regions of the mask were populated with different intensity ratios for each metabolite. Each region was then populated with the intensities for these peaks with normally distributed random fluctuation in intensity. Additional peaks from a blank α-Cyano-4-hydroxycinnamic acid (CHCA) matrix spectrum (a commonly used MALDI matrix) were also added with normally distributed fluctuation in intensity. This dataset contains 30 739 pixels, 1577 m/z channels and mask dimensions of 152 × 307. An example ion image from these data is provided in Fig. 2, available as supplementary data at *Bioinformatics Advances* online.

#### 2.1.2 More homogeneous synthetic MALDI

A second more homogeneous synthetic MALDI dataset was also created. These data simulate biological data with less distinct features because the original synthetic data are well defined with distinct clusters and as such are easily differentiated by most algorithms and hyperparamaters. For this purpose, the mean spectrum of the original synthetic MALDI dataset for the whole dataset (μ_w_) as well as the mean spectrum for each region (μ_r_) were calculated. These were used to create the new intensities for each pixel *p_new_*. Following this, each pixel has a specified percentage (s) of the mean spectrum of its corresponding region subtracted from it, and then the same percentage of the mean spectrum from the whole dataset added.


$$p_{\mathrm{new}}=p_{i}-\;{s\mu }_{r}+{s\mu }_{w}$$

This more homogeneous synthetic MALDI dataset is created of pixels which possess 90% mean spectrum and 10% of the mean spectrum associated with their given cluster. This dataset is comprised of 30 739 pixels, 1577 m/z channels and mask dimensions of 152 × 307.

#### 2.1.3 Disparate synthetic MALDI

The disparate synthetic data simulates a biological challenge whereby one cluster is much greater (many more samples) than others. This is used to exemplify cases such as in the study of diseases, where often healthy tissues are in greater abundance (Angerer *et al.* 2016). One of the masks for the regions in the initial synthetic data was selected, and spectra were randomly selected and duplicated from this region using a bootstrapping approach. In total, 30 739 spectra were created using this method to increase the number of pixels in this region from 6160 to 36 899. The total dataset contains 61 478 pixels, 1577 m/z channels and mask dimensions of 307 × 304.

#### 2.1.4 Synthetic MALDI + more homogeneous synthetic MALDI

This dataset aims to create a greater distribution of data, where there are distinct clusters, in addition to more homogeneous and less defined structures. An appropriate dimensionality reduction will preserve distinct in addition to more subtle relationships when both are present. The more homogeneous synthetic MALDI dataset was concatenated with the original synthetic MALDI dataset, where the mask for the synthetic MALDI dataset is above that of the more homogeneous dataset. The dataset is comprised of 61 478 pixels, 1577 m/z channels and mask dimensions of 307 × 304.

#### 2.1.5 Experimental data

We introduce three biological datasets from murine brain tissue which utilize the most common acquisition methods in MSI, desorption electrospray ionisation (DESI) and MALDI. These cover a range of imaging pixel sizes commonly used in MSI (20 μm–50μm) and are acquired in negative and positive polarities (detection of negative and positive ions produced respectively) across transverse and sagittal sections of mouse brain. Brain tissues were analysed because they have many, well defined anatomical regions and can be compared to reference atlases such as the Allen brain atlas (Jones *et al.* 2009), in addition to comparison to our synthetic datasets. All organs were snap frozen in liquid nitrogen following excision, and tissues were cryosectioned at 10 μm thickness. Tissue sections were then thaw-mounted onto SuperFrost slides, vacuum packed, and stored at –80°C prior to analysis. Samples were warmed up to room temperature in the vacuum-packed container.

#### 2.1.6 DESI analysis of sagittal brain at 50 μm

A DESI source was coupled to a Xevo G2-XS (Waters corporation, UK) mass spectrometer. A 50:50 (%v/v) MeOH/EtOH spray solvent was used with a flow rate of 2 µl/min. Analysis was carried out in negative ion mode, as was mass calibration. For calibration, an in house created polylactic acid (PLA) sublimed slide was used. Data was acquired, with a mass range of m/z 50–1200, a pixel size of 50 µm × 50 µm and stage speed of 100 µm/s. This collects data at 2 pixels/s.

#### 2.1.7 MALDI analysis of sagittal brain at 20 μm

MALDI MSI analysis of the 20 μm sagittal brain sample was acquired on Waters Synapt G2si Q-ToF instruments (Waters, UK) with a prototype uMALDI source. A mass range of m/z 100–1500, 20 scan μm pixel size, scan speed of 400 μm/s, positive ion polarity, and CHCA matrix was used. This dataset contains 123 557 pixels, 2000 m/z channels and mask dimensions of 355 × 748.

#### 2.1.8 DESI analysis of transverse brain at 50 μm

Mouse brain tissue was sectioned from the transverse plane at 12 μm thickness using a cryo-microtome (CM 1850, Leica, Milton Keynes, UK) and thaw mounted onto Superfrost slides (Superfrost Plus, Thermo Fisher, Waltham, MA, USA). DESI MSI data were acquired on a Waters Xevo instrument using a pixel size of 50 μm, scan rate of 200 μm/s, mass range of m/z 50–1200, 95/5 MeOH/H20 solvent in sensitivity mode and negative ion polarity. This dataset has 9287 pixels, 2000 m/z channels, and mask dimensions of 125 × 125.

#### 2.1.9 Data processing

All data pre-processing until the generation of a datacube was performed using SpectralAnalysis (Race *et al.* 2016) or MATLAB scripts, (2017a; Mathworks, USA) after conversion of RAW files to imzML (Chambers *et al.* 2012). The DESI(-) sagittal brain dataset was re-binned with a bin size of 0.001 using an interpolation re-binning method, and mean spectra were generated. For all datasets, a region of interest (ROI) was generated for tissue only data before datacube generation.

#### 2.1.10 t-SNE method

All t-SNE embeddings were generated using the public Sklearn (Fabian Pedregosa *et al.* 2011) python library, sklearn.manifold.TSNE function version 1.4 using the following parameters: n_components = 3, learning_rate = auto, init = random, metric = cosine, all other parameters were default with the exception of perplexity and exaggeration which will be outlined for each dataset (Fabian Pedregosa *et al.* 2011). For dimensionality reduction of MSI data, cosine distance has been found to lead to superior embeddings and as such is the reason for choice in this study (Smets *et al.* 2019). For each dataset described, every combination of perplexity and exaggeration (Supplementary material) was used to create an embedding.

#### 2.1.11 UMAP method

All UMAP (McInnes *et al.* 2020) embeddings were generated using the public umap library using the following parameters: n_components = 3, metric = ‘cosine’, init=’spectral’, all other parameters were default with the exception of n_neighbours where the values used were 2, 3, 4, 5, 10, 15, 20, 25, 30, 50, 100, 200, 300, 400, 500, 750, 1500, 2000, 3000, 5000, 7500, 10000 for the synthetic MALDI and more homogeneous synthetic MALDI datasets.

#### 2.1.12 Dimensional cophenetic integrity

The cluster relationship preservation method (workflow in Fig. 1) predominantly relies on the hierarchical clustering of high and low dimensional space before calculation of similarity between the two clustered relationships. A comprehensive description can be found in supplementary material and can be separated into the following four steps:

##### 2.1.12.1 Sampling of datapoints

**Figure 3:**
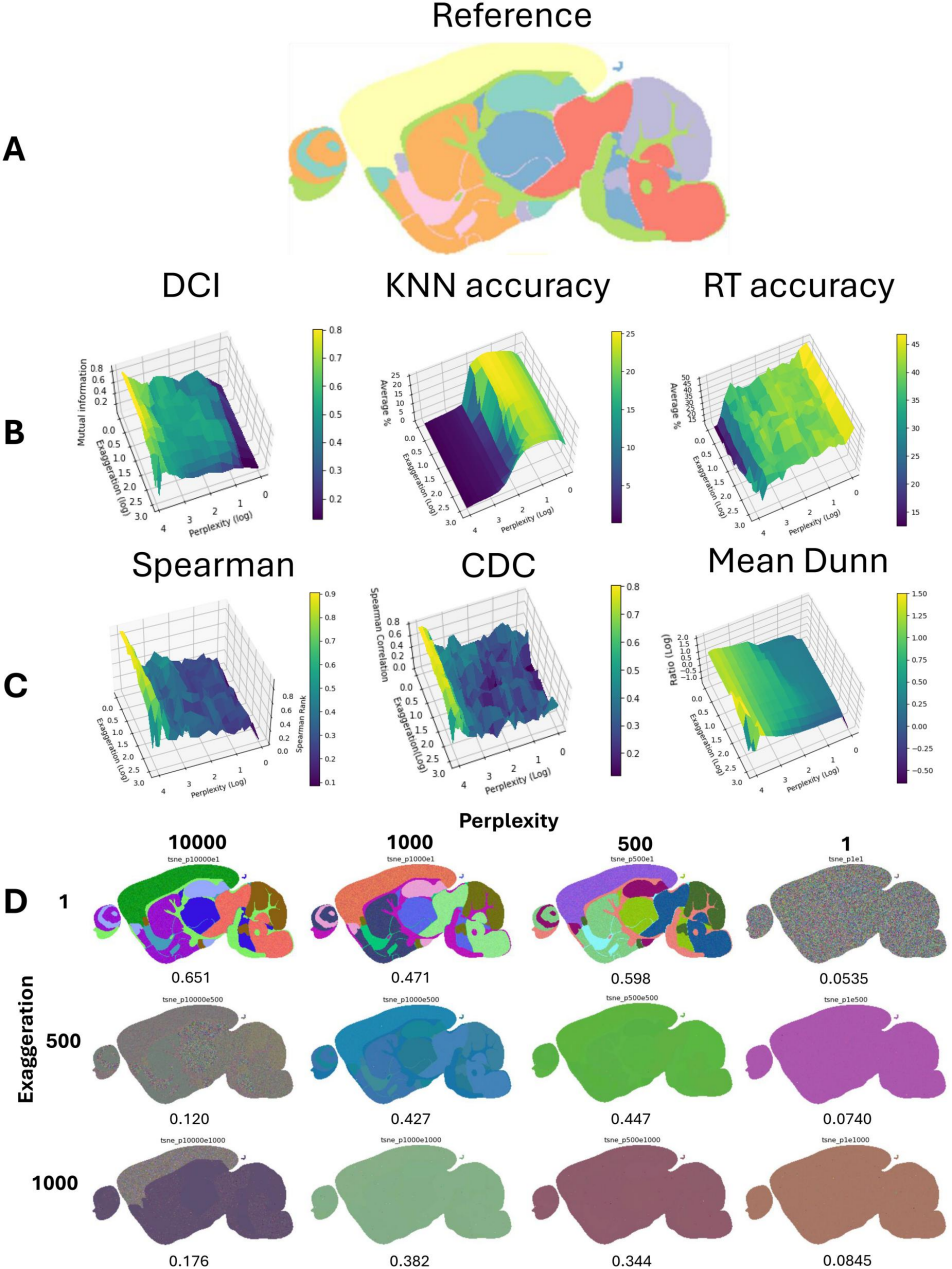
(A) Synthetic MALDI reference image. (B) Gridplot of DCI, KNN accuracy, and RT accuracy for a range of perplexity and exaggeration results, respectively. (C) Gridplot of Spearman rank, Centroid distance correlation, and Inter/Intra distance ratio for a range of perplexity and exaggeration results, respectively. (D) 3D reduced representation embeddings of a range of Perplexity and Exaggeration with their associated DCI results (left to right).

To generate a subset of the total data representative of a variety of present features, kmeans clustering is applied to the low dimensional space with k = n/100, where n = total samples in the data. This number of samples captures the overall differences in DCI scores across the tested datasets whilst decreasing the processing time. DCI score was evaluated across a range of sample numbers (0.5–25% total sample size) and found little difference in scores, so 1% was selected for efficiency (Fig. 3, available as supplementary data at *Bioinformatics Advances* online). For further parameters see supplementary material. The closest data points to each cluster centroid are then taken to give a subset of the total data that is representative of the features present in the whole data. Pairwise distance for the low and high dimensional space were calculated using Euclidean and cosine distances respectively utilising the Scipy python library function scipy.spatial.distance.pdist version 1.13.1 (Virtanen *et al.* 2020). Cosine distance was chosen as it is the metric used in the t-SNE embedding process.

##### 2.1.12.2 Hierarchical clustering of samples in high and low dimensions

The condensed distance matrix for high and low dimensional space are used to generate a linkage matrix (average linkage) using the public library Scipy (Virtanen *et al.* 2020), function scipy.cluster.hierarchy.linkage version 1.13.1 with cosine and Euclidean distance for high and low dimensional space respectively. Use of hierarchical clustering removes the necessity for cluster number determination, which is an area of ongoing study in many fields. This type of clustering also provides layers of relationship granularity through local to global relationships. Whilst use of such methods on high dimensional space will always be affected by the ‘curse of dimensionality’, hierarchical clustering can capture relationships even in high dimensional space. By using an average linkage we produce more stable cluster shapes reflecting overall distributions being less sensitive to outliers, and use of cosine distance removes the assumption of Euclidean geometry.

##### 2.1.12.3 Creation of cophenetic distance matrices based on the hierarchical clustering

Cophenetic distance is a metric which measures the similarity between two data points based on their height (distance) in a hierarchical clustering dendrogram. These matrices were calculated from the linkage matrices using Scipy (Virtanen *et al.* 2020) function scipy.cluster.hierarchy.cophenet version 1.13.1. Use of cophenetic distance provides a quantitative method to measure the relationships across the hierarchical clustering dendrogram, and creation of distance matrices which can be compared across dimensions. This comparison is appropriate because whilst the high dimensional space may be more abstract/noisy, cophenetic distance summarizes overall cluster relationships, as opposed to singular pointwise relationships, making this a robust method to evaluate the preservation of relationships across dimensions.

##### 2.1.12.4 Mutual information calculation between high and low dimensional cophenetic distance matrices

An arithmetic mean normalised mutual information, was calculated between condensed high and low dimensional cophenetic distance matrices using Scikit-learn (Fabian Pedregosa *et al.* 2011) function sklearn.metrics.normalized_mutual_info_score version 1.4 with default parameters. Mutual information does not assume linearity in relationships, and can be used in the presence of heteroskedasticity which makes this method a robust measure of correlation between the high and low dimensional cophenetic distance matrices.

This cluster preservation method, referred to herein as dimensional cophenetic integrity (DCI), is implemented similarly across all datasets described in this work.

#### 2.1.13 Bayesian optimization

The DCI score can form the objective criterion for an optimisation algorithm such as Bayesian optimisation or an evolutionary algorithm. To demonstrate a proof of concept for this approach we have performed Bayesian optimisation with DCI as the optimisation criterion, and perplexity and exaggeration t-SNE hyper-parameters as the search space. This was selected because it is widely used in many fields, is fast and is robust to changes in parameters (Jalas *et al.* 2021, Yu *et al.* 2023). The publicly available scikit-optimize python library (Head T *et al.* 2024) version 0.10.2 and gp_minimize function was used with the following parameters: func = dci, dimension = search_space where search_space is a list of perplexity (range 5, 10000) and exaggeration (range 1, 1000) values of type integer using skopt.space, n_calls = 120, n_initial_points = 60, random_state = 42.

## 3 Results

The hyperparameters chosen for all embeddings used to assess each dimensionality reduction evaluation method are ‘perplexity’ and ‘exaggeration’. These are commonly regarded as the most important or commonly altered (from default values) parameters of the t-SNE dimensionality reduction algorithm. There are many parameters of this algorithm that can be changed and which would affect the process of the dimensionality reduction, however it will be shown that with careful selection of only these two, the cluster preservation of data can be optimised for this most simple case of parameter selection. It is important to note that although t-SNE is used to demonstrate DCI performance, any dimensionality reduction algorithm and combination of hyperparameters can be evaluated due to the fundamental way in which DCI evaluates cluster relationship preservation. DCI results for a series of UMAP embeddings are shown to compare embedding performance to t-SNE.

### 3.1 Transverse DESI results

We first compare the DCI results with the local and global evaluation methods, k-nearest neighbour (KNN) accuracy, Spearman correlation and random triplet (RT) accuracy (Fig. 2). Random triplet and KNN accuracy are percentage-based scores whereby 100% accuracy constitutes the best-case score, whereas mutual information is an arithmetic mean normalized measure where 1 is the greatest score of similarity. CDC and the mean based Dunn methods cannot be used here as we have no labelled reference with which to compare.

DCI produces higher scores for embeddings with visually higher quality of tissue segmentation when comparing perplexity 7500 exaggeration 1 and perplexity 1 exaggeration 1, which is expected when preservation of structural and pattern relationships of data is maintained from high to low dimensional space (Fig. 2C). Higher perplexity and lower exaggeration lead to higher DCI scores, and therefore better preservation of data from high to low dimensional space. The KNN accuracy and RT accuracy results show the opposite of DCI, lower perplexity values lead to higher scores. Notably, both RT and KNN accuracy are not indicative of visual quality of tissue segmentation (Fig. 2 panel A, B right, Perplexity 1 exaggeration 1, Perplexity 500, exaggeration 1000). KNN accuracy is a measure of local structure preservation but does not capture global structure, and RT accuracy is a measure of global structure preservation but does not preserve local information. Therefore, both metrics do not evaluate the overall preservation of manifold structure to low dimensional space. Spearman correlation has comparative results to DCI, however DCI shows maximum embedding quality at ∼log 3 perplexity, whereas the Spearman correlation has increasing scores up to log 4 perplexity.

Other than visual quality of tissue segmentation, it is impossible to assess whether DCI or pairwise Spearman’s correlation is performing most optimally as labels do not exist for this real biological dataset and the true sample relationships are not known. It is for this reason that we introduce a synthetic MALDI dataset where these relationships are known to benchmark these evaluation metrics.

### 3.2 Synthetic MALDI results

Use of a synthetic dataset provides a ground truth to study the DCI results, as the visual performance should reflect the true structure of the data. This synthetic MALDI dataset represents a simple data structure, with 8 well defined clusters. These clusters have distinct mean mass spectra based on the sagittal plane of the Alan brain atlas (Jones *et al.* 2009). The clearly defined clusters of this dataset provide a benchmark for DCI, as the dimensionality reduction algorithm should perform well, producing an embedding with high preservation of cluster relationships.

In addition, it allows us to test and compare the local and global unsupervised dimensionality reduction evaluation methods previously published by Huang et al with DCI, as a reference image exists of which comparisons can be made (Huang *et al.* 2022).

CDC appears to have noisy results up to log 3 perplexity, but shows relatively similar results between Spearman correlation, and the mean based Dunn index. In this case, all methods could be used to produce similar results, however CDC requires labelling and Spearman takes notably longer to run.

**Figure 4:**
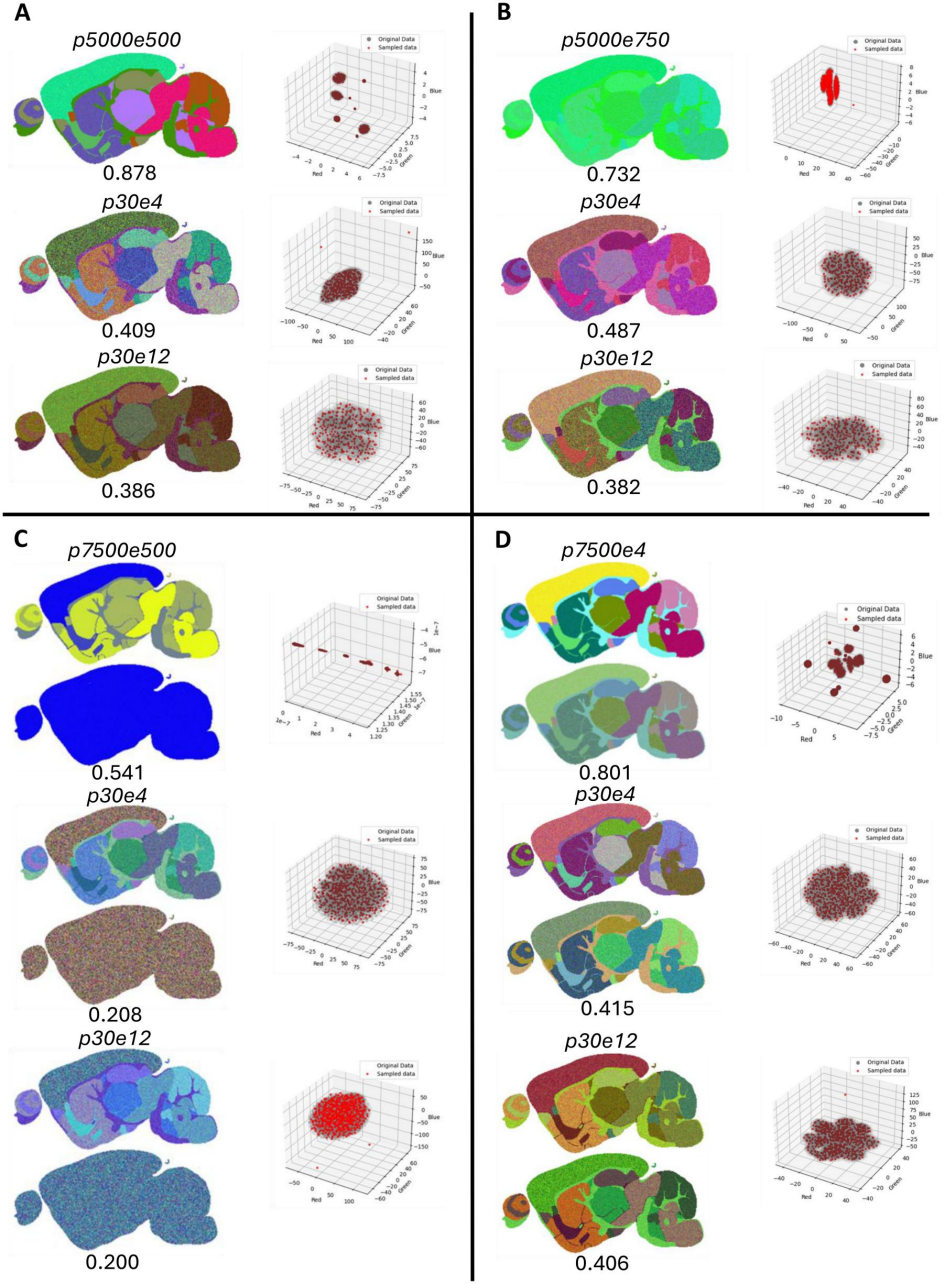
(A–D) highest quality embedding according to DCI which has the greatest mutual information, p30e4 embedding which is the van der Maaten recommended hyper parameters, and p30e12 embedding which is the Sklearn default hyperparameters, top to bottom per panel, for synthetic MALDI, more homogeneous synthetic MALDI, disparate synthetic MALDI and synthetic + more homogeneous synthetic MALDI respectively. Associated scores under the embedding, and 3D scatters to the right.

A generalisable method must work across multiple DR algorithms for consistent evaluation across studies. We therefore evaluate DCI amongst the other discussed methods on UMAP embeddings and can be seen in Fig. 4, available as supplementary data at *Bioinformatics Advances* online. The UMAP embedding produced with 2 neighbours appears visibly noisy, and has a low mean based Dunn index, as well as DCI, however in this case, use of Spearman’s correlation of pairwise distances is not suitable as it produces a high score despite the poor embedding according to the mean Dunn index and visual comparison. This demonstrates DCI score is indicative of the quality of the embedding which is not always the case for Spearman’s correlation of pairwise distances.

**Figure 5:**
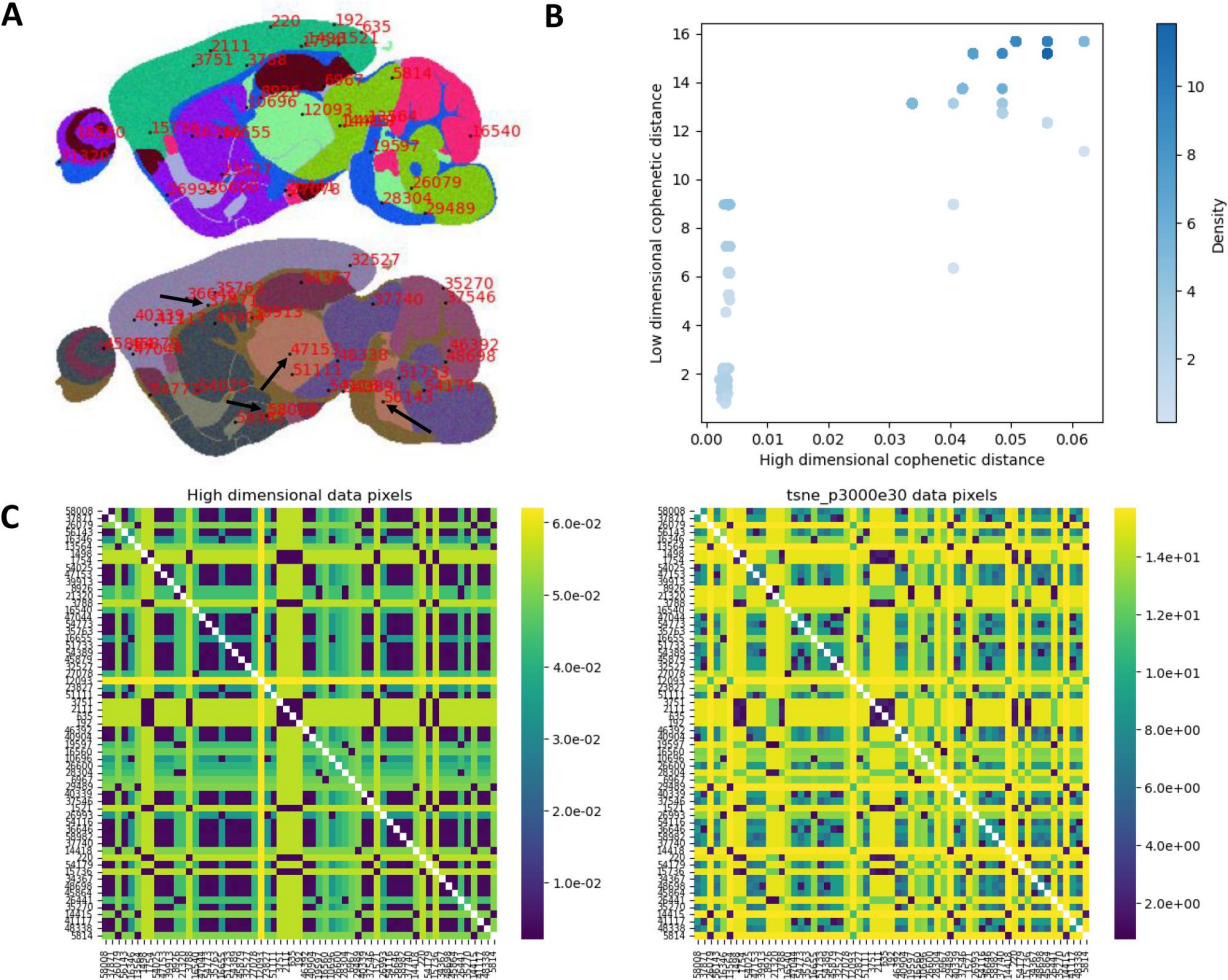
(A) A t-SNE embedding of the synthetic MALDI + more homogeneous synthetic MALDI dataset using perplexity 3000 and exaggeration 30. Black arrows point to pixels 47153, 58008, 37871, and 56143. (B) A scatter plot of the t-SNE p3000e30 cophenetic distance Versus the high dimensional cophenetic distance. (C) The pairwise cophenetic distance matrix between sampled points for the high dimensional space (left), and the t-SNE embedding (right).

For DCI, higher values of perplexity, and lower values of exaggeration lead to higher quality embeddings, which is shown by the higher mutual information scores, as well as the embeddings looking visually more similar to the reference image. Changing the values of exaggeration appear to affect DCI results the most compared to the effects of changing values of perplexity. The general trend is that larger values of perplexity, and smaller values of exaggeration, lead to greater cluster preservation results according to DCI. The embeddings produced with perplexity 1000 and exaggeration 500, and perplexity 500, exaggeration 500 (Fig. 3B, left) show some similarities in structure to the synthetic data reference image. However, the reason for these embeddings producing homogeneous looking images is the creation of outliers from the dimensionality reduction; some samples (pixels) have been inappropriately embedded with poor structural preservation. Further embeddings and their associated scores for ‘low’, ‘medium’, and ‘high’ scores can be seen in Fig. 5, available as supplementary data at *Bioinformatics Advances* online.

KNN and RT accuracy provide information on the topological structure of the data on average, however, do not provide any information on the correlation between features, or interpretability of clusters. More importantly, these results are not indicative of the quality of dimensionality reduction when compared to the known regions in the synthetic data. This is evident by the high scores of KNN and RT accuracy even for poor tissue segmentation (Fig. 3D, Perplexity 1 Exaggeration 1, Perplexity 1, exaggeration 500, Perplexity 1, Exaggeration 1000). Evaluation of pattern and data structure preservation by DCI incorporates the assessment of sample similarity across a variety of sample distances, including local, global and ‘mid-range’ points and therefore provides a better evaluation of overall data structure preservation from high to low dimensional space.

**Figure 6:**
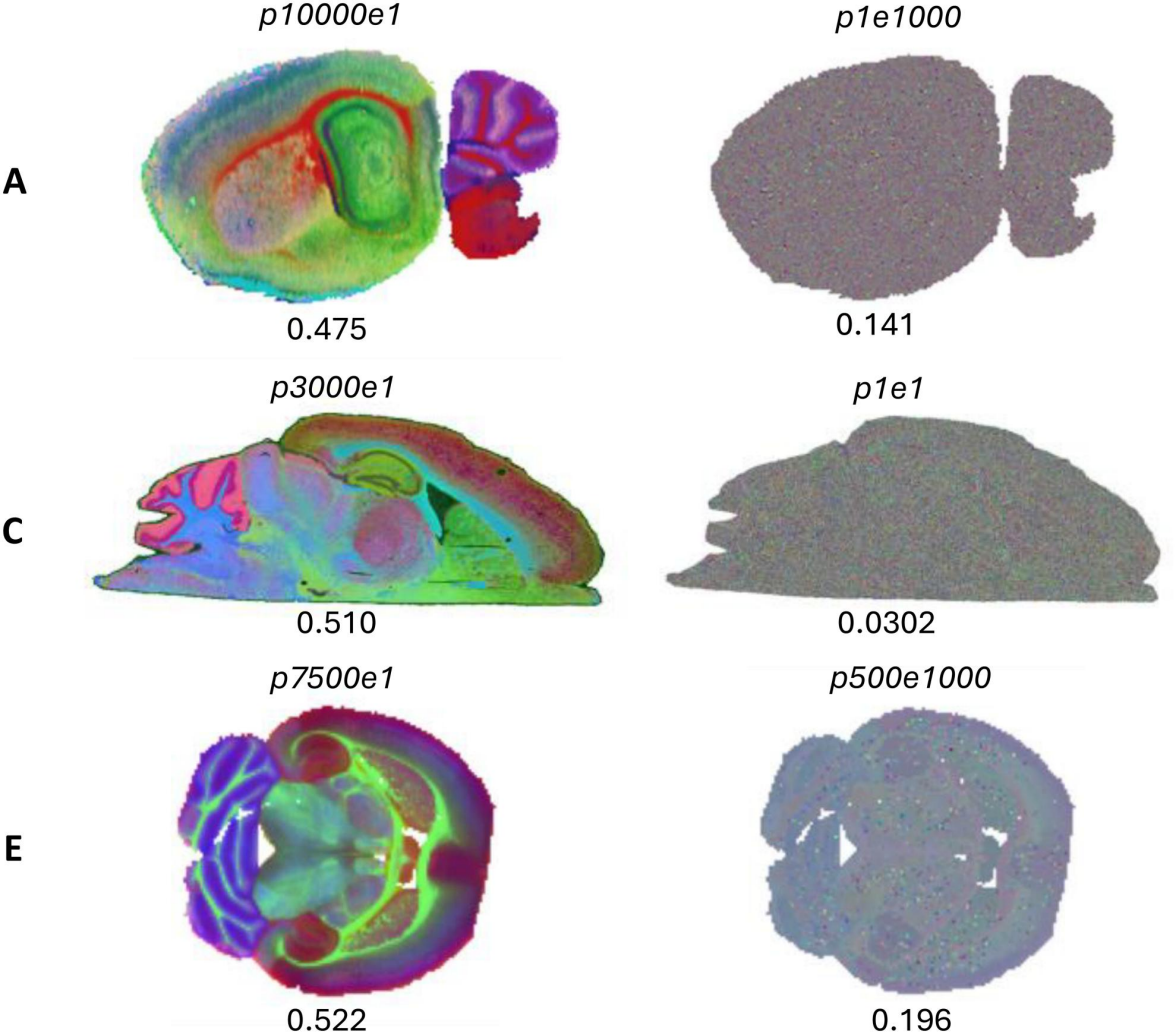
(A–C) Example higher quality embeddings (left) and lower quality embeddings (right) for the 50 μm sagittal brain dataset, 20 μm MALDI dataset, and 50 μm transverse brain dataset respectively. Associated DCI scores for each embedding are specified below.

The use of PCA initialization for t-SNE does not produce significantly different results when compared to use of a random initialization (Fig. 6, available as supplementary data at *Bioinformatics Advances* online). This is expected since PCA is typically used prior to tSNE primarily to speed up the computation rather than to improve the embedding itself.

Next, we use DCI to assess the preservation of cluster relationships in a series of synthetic and real biological datasets with various challenges in data structure. The optimum embedding according to DCI is shown compared to the embeddings generated by Sklearn (Fabian Pedregosa *et al.* 2011) and van der Maaten default hyperparameters (van der Maaten and Hinton 2008).

In MSI, default t-SNE hyperparameters are commonly employed either from the Sklearn (Fabian Pedregosa *et al.* 2011) implementation, or Matlab (Smets *et al.* 2019). Additionally, some MSI studies reference the original t-SNE publication in 2008 by L van der Maaten and Geoffrey Hinton for using their t-SNE implementation (van der Maaten and Hinton 2008, Smets *et al.* 2019, Goossens *et al.* 2022). The produced t-SNE embeddings for the Sklearn (Fabian Pedregosa *et al.* 2011) (perplexity 30 exaggeration 12) and van der Maaten (van der Maaten and Hinton 2008) (perplexity 30 exaggeration 4) suggest default hyperparameters lead to poor quality embeddings both visually and according to DCI, (Fig. 4) for each synthetic dataset. In comparison, the hyperparameters that produce the embedding with the highest DCI score give visually well differentiated segments. The results of this analysis shows that use of default parameters can lead to sub-optimum t-SNE embeddings, and thus this evaluation method helps the user to choose parameters leading to embeddings which preserve the underlying pattern of the data and cluster preservation the greatest.

For the synthetic MALDI dataset, this method of evaluating cluster preservation leads to the identification of an embedding with the greatest mutual information of 0.878 from the embedding with perplexity 5000, exaggeration 500 (Fig. 4A). This represents a mutual information of more than 2× larger than the Sklearn (Fabian Pedregosa *et al.* 2011) default and the van der Maaten implementation which also looks visibly noisier. Typical default perplexity and exaggeration values range between 5–50 and 4–12 respectively. This suggests that users of t-SNE using default hyperparameter ranges without use of an objective evaluation criteria would not have created the embedding with the greatest cluster preservation.

**Figure 7:**
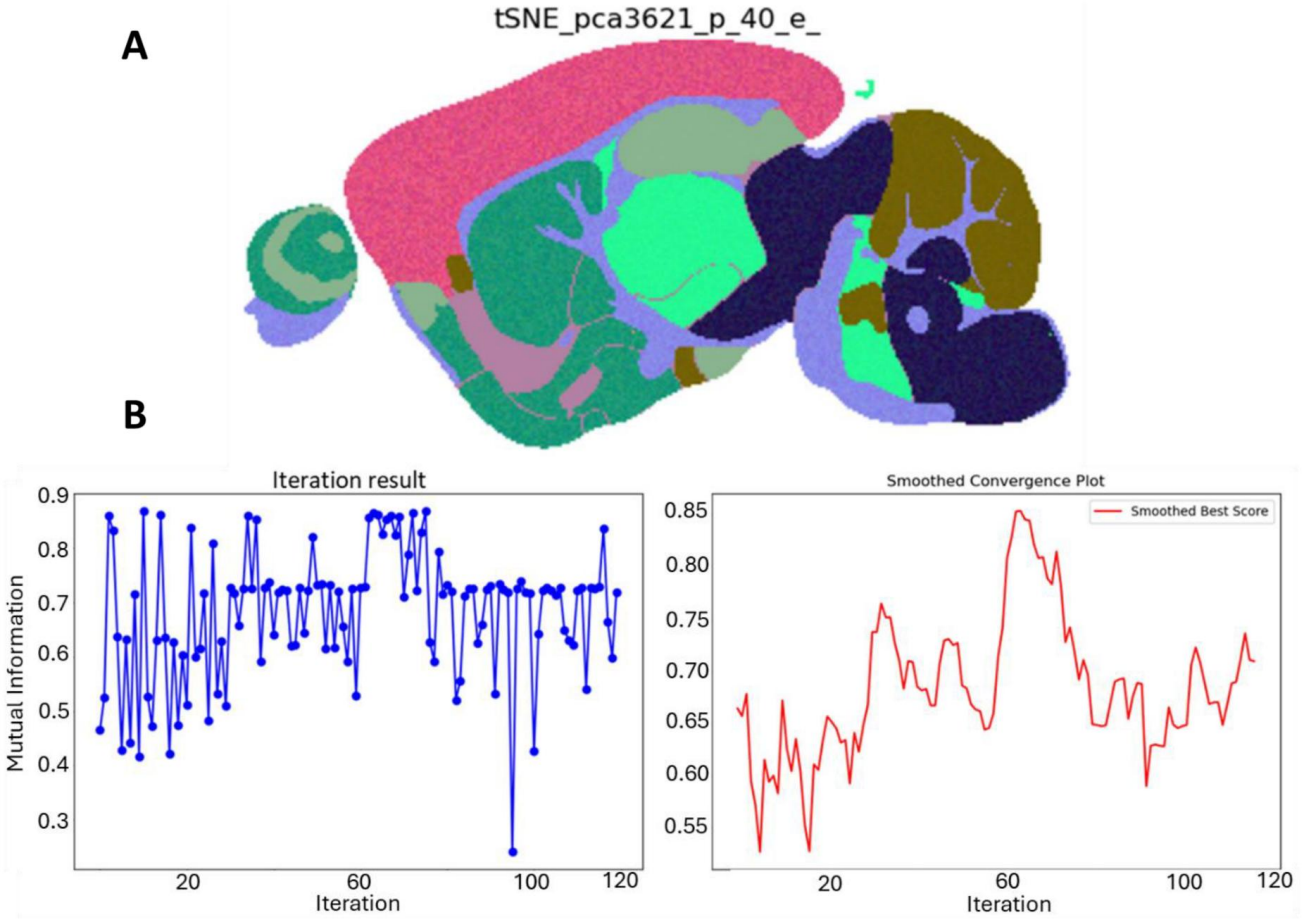
(A) The greatest embedding found by Bayesian optimization using DCI. (B) Left shows the absolute mutual information by DCI for each embedding generated by every iteration of the Bayesian optimization. Right shows a moving average of the mutual information for each iteration of optimization using a sliding window of 5.

This dataset is well defined with clearly distinguished clusters, which may be the reason for the large differences between embedding quality with different hyperparameter selection. DCI results of further synthetic datasets, with unique differences in data structure to simulate various challenges in biological analyses, can be found in the supplementary material. For each synthetic dataset, ‘low’, ‘medium’, and ‘high’ DCI scores and their representative embedding can be seen in Figs. 5, 8, 11, and 14, available as supplementary data at *Bioinformatics Advances* online. The overview of embedding mutual information for different hyperparameter combinations for each synthetic dataset are similar to that of the synthetic MALDI dataset, and can be seen in Figs. 7, 9, and 13, available as supplementary data at *Bioinformatics Advances* online. Generally, a pattern of increased perplexity and decreased exaggeration is observed, leading to greater mutual information.

### 3.3 Disparate regions sizes

The first example of a biological challenge is a dataset possessing a disparately large cluster (many more pixels in one cluster compared to all others), or group of similar data compared to other clusters. Relative distances between datapoints of disparate cluster sizes can become distorted when pairwise similarities are modelled.

The decreased DCI scores (Fig. 7, available as supplementary data at *Bioinformatics Advances* online) for this dataset clearly highlight the well-known issue t-SNE has with preserving global features, as it is an algorithm which favours local feature preservation. The inclusion of this larger cluster has visually made the comparatively smaller clusters of data appear more ‘noisy’, which is likely due to the crowding problem faced by dimensionality reduction algorithms (more information in Fig. 7, available as supplementary data at *Bioinformatics Advances* online). UMAP generated embeddings with greater DCI scores at lower values of neighbours. Additionally, UMAP created a more optimal embedding at 5000 neighbours (Fig. 9, available as supplementary data at *Bioinformatics Advances* online).

### 3.4 More homogeneous data

The homogeneous synthetic MALDI (Fig. 10, available as supplementary data at *Bioinformatics Advances* online) dataset aims to represent a more likely scenario in the analysis of biological tissues, where the relationships between clusters are not very distinct. The main difference is lower DCI scores which are indicative of a decrease in cluster relationship preservation compared to the synthetic MALDI dataset. This could be due to a decrease in the relationship preservation between distant or close datapoints which will be discussed herein.

Performing t-SNE with the suggested perplexity and exaggeration recommended by van der Maaten (p30e4) or default values from Sklearn (Fabian Pedregosa *et al.* 2011) (p30e12) produces a poor cluster relationship preservation and mutual information scores of 0.487 and 0.382 respectively in comparison to well defined clusters and a high mutual information score of 0.732 with optimised hyperparameters of p5000e750 (Fig. 4B); It can be seen that the p5000e750 embedding looks visually homogeneous, and the 3D scatter appears to have clusters close together, however this is due to an anomalous sample (pixel) in the t-SNE representation. This embedding with anomalous pixels removed can be seen in Fig. 11, available as supplementary data at *Bioinformatics Advances* online. Removal of such outliers produces an embedding with visually distinct clusters, which shows that use of DCI is robust against such few outliers.

Mutual information scores are indicative of embedding similarity to the reference regions of the dataset which provides further confidence in the consistency of DCI. The cophenetic distance matrices produced by this method can be inspected to understand the differences in how relationships between distant and close datapoints are preserved. For this dataset, ‘mid-near’ relationships appear to dominate high dimensional space but are poorly preserved in low dimensional space which can be seen in Fig. 12, available as supplementary data at *Bioinformatics Advances* online. t-SNE uses a student t-distribution for pairwise similarity calculations in low dimensional space to alleviate the crowding problem (van der Maaten and Hinton 2008). The heavy tails of the student t-distribution allow t-SNE to preserve distant points which were in high dimensional space, to low dimensional space. This heavy tail causes mid-near points to be exaggerated and pushed further apart as they have a higher probability of having greater distances.

Spearman correlation and the centroid distance correlation score increase with perplexity; perplexity 1000 exaggeration 1 is a noisy embedding with a low DCI and mean Dunn score, however this remains a high score for Spearman correlation. DCI shows a local maxima at ∼log 3 perplexity which is similar to that of the mean based Dunn index showing that these embeddings preserve the relative differences in distance between local clusters and separate cluster centroids. As with the synthetic MALDI dataset, Spearman correlation remains relatively stable across all embeddings, with the noisy low quality 2 neighbour UMAP embedding having a similar spearman correlation to that of a less noisy, higher quality 50 neighbour UMAP embedding (with a greater DCI score) seen in Fig. 12, available as supplementary data at *Bioinformatics Advances* online.

### 3.5 Varying data relationships

This final synthetic dataset combines the synthetic MALDI dataset and the more homogeneous synthetic MALDI dataset. This represents a biological scenario whereby clusters or groups of data are related to other clusters by sharing a certain amount of similar characteristics. For this dataset, each cluster in the synthetic MALDI dataset share 10% of the same mean spectra to its respective cluster in the more homogeneous synthetic MALDI dataset. These clusters should be separate and distinct from each other, but with their relationship preserved.

DCI can be used to identify more detailed relationships in the data by identifying sample relationships from the cophenetic distance matrix and visualising them. To demonstrate this, a lower sub sample of data points are used for this purpose (Fig. 5).

Relative cophenetic distances have been maintained well between the high dimensional cophenetic distance matrix and the t-SNE reduced representation cophenetic distance matrix (Fig. 5B). There appear to be two distinct groupings of data, being localized around low distances, and high distances which can be attributed to the synthetic dataset containing distinct clusters. Additionally, t-SNE exaggerated distances which is the reason for the greater range of distances where high dimensional distance is low. Some samples have not preserved their relative cophenetic distances from other samples in the t-SNE embedding, which can be found upon inspection of Fig. 5C. In the high dimensional cophenetic distance matrix, pixel 47153 has a very small cophenetic distance to all other labelled pixels. However, in the t-SNE embedding, pixel 47153 has the same relative distance to pixel 56143, but has a greater relative distance to pixels 58008 and 37871. As these pixels originate from the more homogeneous portion of this dataset where each pixel contains 90% of the mean spectrum, thus all of these pixels should have small cophenetic distances considering their close relation. It is evident that in the embedding process, t-SNE has exaggerated the differences between pixels which works well for maintaining relative distances between very close or very distant samples, however preservation of relationships with ‘medium’ distances are not optimally preserved. This ‘exaggeration’ of relationships is also visualised in the scatter plot of Fig. 15D, available as supplementary data at *Bioinformatics Advances* online. Some points which are close in high dimensional space, have been pushed away in feature space within the low dimensional embedding. This has also been done to points with distant relationships. More information can be found in section: More homogeneous data.

The exaggeration of sample distances is a significant observation as t-SNE dimensionality reduction before clustering is common in MSI studies; this can be misinterpreted as certain clusters not being as closely related as they should be, potentially missing significant biological relevance between features. This demonstrates a useful application of DCI, which is the ability to identify the relationships between clusters. DCI evaluation leads to the creation of the most optimal embedding with a cluster preservation approximately 2× greater than the one obtained with default van der Maaten and Sklearn (Fabian Pedregosa *et al.* 2011) parameters which provided poor embeddings. These can be seen in Fig. 4D.

### 3.6 Translation to real datasets

After benchmarking DCI against different synthetic datasets, hyperparameter combination for optimum preservation of cluster relationships in real datasets is presented.

We have evaluated the performance of these hyperparameters in a range of MALDI and DESI datasets in positive and negative ion mode from different orientations of mouse brain. The relationship between hyperparameter combination and embedding quality is similar to that of the synthetic datasets with larger perplexity values leading to greater mutual information. An overview of the DCI for these different datasets can be seen in Fig. 2, Figs. 15 and 18, available as supplementary data at *Bioinformatics Advances* online. A range of embedding quality with various hyperparameter combinations can also be seen in Figs. 16, 19, and 21, available as supplementary data at *Bioinformatics Advances* online. DCI consistently found the optimum embedding with higher quality than the van der Maaten and Sklearn (Fabian Pedregosa *et al.* 2011) hyperparameters, where the Sklearn hyperparameters always lead to a poorer quality embedding than those of van der Maaten as shown in Fig. 17, 20, and 22, available as supplementary data at *Bioinformatics Advances* online. Exemplar images of each real dataset of higher quality embeddings with higher DCI scores, and low-quality embeddings with low DCI scores are shown in Fig. 6.

### 3.7 DESI sagittal brain

Unlike the synthetic MALDI dataset, the correlation scores for this dataset are generally much lower, which is to be expected considering the lack of clearly distinct clusters or groups of data with a real biological sample. The disparate synthetic MALDI dataset has comparable mutual information scores (0.533 for perplexity 7500 exaggeration 500, with scores between 0.2 and 0.5), and it can be seen within the highest scoring embedding images that there does exist a larger similarly related group of samples (Green in p1000e1 Fig. S16, available as supplementary data at *Bioinformatics Advances* online) than other pixels and could indicate that this is the reason for the lower scores. This could be indicative of global features being more important to preserve, with higher values of perplexity aiding in doing so.

### 3.8 20 μm MALDI sagittal brain dataset

As with other synthetic and biological datasets, higher values and perplexity and lower values of exaggeration lead to greater DCI scores and preservation of cluster relationships (Fig. 19, available as supplementary data at *Bioinformatics Advances* online). This 20 μm dataset is the largest dataset analysed within this work, comprising of ∼120 000 pixels and 2000 m/z channels. A large grid search was not feasible and therefore a ‘coarse’ and ‘fine’ search was conducted. For this dataset, a maximum perplexity of 3000 was used as larger values would cause our process to run out of available RAM (more details in supplementary material section 20 μm MALDI sagittal brain).

A limitation of such large grid searches which have been used in this work is the large computational resources required to generate so many embeddings and using such high values of perplexity. This is incredibly time consuming to do, and something that could be optimised by use of other methods such as evolutionary algorithms or Bayesian optimization which has been demonstrated within this work (Embedding optimization using DCI).

### 3.9 Transverse brain dataset

DCI found the most optimum embedding for perplexity1500, exaggeration50, with a mutual information score almost 2× larger than the two embeddings obtained with van der Maaten and Sklearn (Fabian Pedregosa *et al.* 2011) when using their default values (Fig. 23, available as supplementary data at *Bioinformatics Advances* online). This showcases the method’s consistent ability to identify the embedding with the greatest preservation in relative cophenetic distances between the original high dimensional space and produced low dimensional representation. The scores obtained in this dataset are more similar to the homogeneous synthetic MALDI dataset, with the transverse brain dataset being similar in size and the biological data itself being more homogeneous without clear distinction between groups of data/clusters. This similarity provides further confidence that the DCI method can reliably identify embeddings with the highest feature pattern preservation even with data which is not clearly defined.

The trend of scores across DCI, KNN accuracy, RT accuracy, and Spearman correlation for UMAP embeddings, are similar to that of the synthetic datasets. Generally, DCI increases with neighbours, and KNN, and RT accuracy decreases. Notably, Spearman correlation for low quality embeddings, such as that for 2 neighbours has a comparably high score to visually superior embeddings (supplementary information S23).

### 3.10 Embedding optimization using DCI

We briefly demonstrate the ability for DCI to be used as an objective criterion for optimization of t-SNE hyperparameters. We demonstrate this using a Bayesian optimization on the synthetic MALDI dataset using scikit-optimize (Head *et al.* 2024) (further details in methods). This optimization quickly identified that high values of perplexity and low values of exaggeration lead to t-SNE embeddings with the greatest mutual information score as generated by DCI. The most optimum embedding found (perplexity 3621, exaggeration 40) with a mutual information of 0.864 in addition to optimization iteration plots can be seen in Fig. 7.

Use of DCI as an objective criterion could help to generate the most optimum embedding without a computationally expensive grid search. This criterion is compatible with alternative optimization approaches such as such as evolutionary algorithms (Coletti *et al.* 2020). Use of Bayesian optimisation has advantages for optimisation of hyperparameter values when performing dimensionality reduction as it has great efficiency and typically requires fewer iterations (Wang *et al.* 2019, Chhantyal *et al.* 2025) to produce optimal result than other methods such as evolutionary algorithms. The efficiency of this optimisation also comes from the exploratory and exploitative nature of the process as the model will sample where it predicts high performance, yet also sample in areas of uncertainty.

Additionally, using DCI as an objective criterion for dimensionality reduction optimization reduces subjective bias from a user generating embeddings, as well as ensuring parameter combinations are evaluated that may not be in a grid search.

## 4 Conclusion

The results shown across a variety of synthetic and none-synthetic datasets demonstrate DCI’s ability to consistently identify embeddings which preserve the relative high dimensional cophenetic distances between samples the greatest. These results are confirmed upon visual inspection of synthetic results when compared to the known reference image, as well as when comparing the patterns between high and low dimensional cophenetic distance matrices for the none-synthetic datasets. Additionally, it has been demonstrated how this method can be used to identify samples which have a good preservation in cophenetic distance relationship, as well as those which have poorly preserved their relative relationships by comparing the labelled samples pixels to a labelled cophenetic distance matrix.

The embeddings with the greatest cluster preservation across the different datasets have perplexity values in line with the expected importance of global relationships. That is, large datasets with many pixels and datasets with disparate clusters produce embeddings with the greatest cluster preservation when using larger values of perplexity. This observation provides further confidence in this method’s ability to evaluate the preservation of cluster relationships in t-SNE reduced representations of data.

The importance of not relying on default hyperparameters choices has also been demonstrated, where the greatest embedding identified by this method has consistently outperformed the embeddings using default hyperparameters in synthetic and none synthetic datasets. It is important to note that only the most common hyperparameters perplexity and exaggeration were used in this work as they are the most used within the MSI community, however other tuneable parameters exist that would affect the outcome of the t-SNE embedding process such as learning rate and initialisation. The benefit of using DCI is that it can be used for all combinations of hyperparameters and is not only limited to those used in this work. Not only can this method be used to optimise hyperparameter choice, but it also provides a way to evaluate new dimensionality reduction algorithms to be used in MSI and other fields.

## Supplementary Material

vbag100_Supplementary_Data

## Data Availability

The data underlying this article is available in Metabolights at https://www.ebi.ac.uk/metabolights/ and can be accessed with the unique identifier: REQ20250618211289. All code can be found on our GitHub: https://github.com/NiCE-MSI/Dimensional-Cophenetic-Integrity-DCI
