# Supplementary material for: Dimensional cophenetic integrity: a method for evaluation of dimensionality reduction in MSI
Source: Bioinform Adv. 2026 Apr 15;6(1):vbag100. doi: 10.1093/bioadv/vbag100 (PMC13110009; doi:10.1093/bioadv/vbag100)
Supplement: vbag100_Supplementary_Data [file vbag100_supplementary_data.zip › Dimensionality reduction paper supplementary 06-01-2026_clean.docx]

**Supplementary information**

**Methods to evaluate DR Literature:**

The use of labels for biological MSI data is not usually possible, and thus classification error cannot usually be used for the assessment of dimensionality reduction evaluation in MSI. Without use of labels, the most intuitive way to assess the quality of dimensionality reduction is to evaluate the preservation of distances between data points from the high dimensional space to the reduced representative (low dimensional) space. It is for this reason that many dimensionality reduction evaluation methods rely on some form of distance measurements and calculation of relative distance preservation between high and low dimensions. A summary of such evaluation methods can be found in table 2.

| Evaluation metric | Overview of method | Use in fields |
| --- | --- | --- |
| Trustworthiness | A reduced representation of data is trustworthy if the k nearest neighbours of a given sample/ data point in high dimensional space are also neighbours in low dimensional space.  $k=1-\frac{2}{Nk\left( 2N-3k-1 \right)}\Sigma_{i=1}^{N}\sum_{j\in U_{k}(i)} (r\left( i,j \right)-k)$  Where N is the number of samples, $r\left( i,j \right)$ is the rank of sample j ordered according to distance from i in high dimensional space and $U_{k}(i)$ are samples in a neighbourhood of size k of sample i in low dimensional space but not in high dimensional space. | Machine learning^1^ Remote sensing, Earth Observation^2^ |
| Continuity | Continuity works the same was as trustworthiness but $V_{k}(i)$ is the sample points in a neighbourhood of sample i in the high dimensional space but not in low dimensional space. $\hat{r}\left( i,j \right)$ is the rank of sample j ordered according to distance from i in low dimensional space.  $k=1-\frac{2}{Nk\left( 2N-3k-1 \right)}\Sigma_{i=1}^{N}\sum_{j\in V_{k}(i)} (\hat{r}\left( i,j \right)-k)$ | Machine learning^1^ Remote sensing, Earth Observation^2^ |
| Spearman’s rho | The correlation of pairwise distance rank order is calculated between high and low dimensional spaces.  $s_{R}=1-\frac{6\sum d_{i}^{2}}{n\left( n^{2}-1 \right)}$  $ⅆ_{i}$ is the rank order difference between each n observation. | Statistics^3,4^ |
| Distance matrix | From a matrix of data, a new pairwise distance matrix can be computed whereby each entry ($Dⅈj)$ is given by $Dⅈj=\left\Vert xi-x_{j} \right\Vert$ where $\left\Vert xi-x_{j} \right\Vert$ is a distance or similarity between points $xi$ and $xj$. These can be made for high and low dimensional data before measurement of correlation between both pairwise distance matrices using a correlation metric such as Pearson correlation. | Machine Learning^5^ |
| Random triplet accuracy | A sample of $N$ triplets (3 data points) are calculated and the relative distances between datapoints within a triplet are measured in high dimensional space. The relative distances between the same N triplets in low dimensional space are measured, and the random triplet accuracy is the percentage of triplets whose relative distances are preserved between high and low dimensions. | Bioinformatics/ Transcriptomics^6,7^ |
| Unsupervised local evaluation method – *Huang et al.* | The average intersection between $N\left( i \right)$ and $N^{'}\left( i \right)$ is calculated across an entire dataset where:  $N\left( i \right)$ is the set of k nearest neighbours for each sampled data point in high dimensional space and $N^{'}\left( i \right)$ is the set of k nearest neighbours in the low dimensional space. | Bioinformatics/ Transcriptomics ^6^ |
| Centroid Distance Correlation (CDC) | Cluster centroids are calculated in high and low dimensional space. CDC is the spearman rank between these high and low dimensional centroids pairwise distances. | Bioinformatics/ Transcriptomics^6,8^ |
| Mean based Dunn index | The ratio of the mean inter and intra cluster centroid distances in either high or low dimensional space. | Machine Learning^9^ |

Table 1 Overview of previously published methods of evaluating dimensionality reduction algorithms

For more information on the evaluation methods described in the table, as well as further methods in dimensionality reduction evaluation, papers by *zhang, shang, zhang,* and *lee, verleysen* have fantastic reviews. ^5,10^

| Citation | t-SNE parameters |
| --- | --- |
| Application of clustering strategy for automatic segmentation of tissue regions in mass spectrometry imaging^11^ | Sklearn default |
| A Machine Learning-Driven Comparison of Ion Images Obtained by MALDI and MALDI-2 Mass Spectrometry Imaging^12^ | Matlab default but with “exact” distance |
| Evaluation of Distance Metrics and Spatial Autocorrelation in Uniform Manifold Approximation and Projection Applied to Mass Spectrometry Imaging Data^13^ | van der Maaten default |
| Data-driven identification of prognostic tumor subpopulations using spatially mapped t-SNE of mass spectrometry imaging data^14^ | van der Maaten default |
| Spatially resolved metabolomic characterization of muscle invasive bladder cancer by mass spectrometry imaging ^15^ | Matlab default |
| Fast interpolation-based t-SNE for improved visualization of single-cell RNA-seq data^16^ | Fit-SNE implementation, perplexity 50, exaggeration 12 |

Table 2 Example t-SNE hyper-parameter combinations for a selection of t-SNE uses in mass spectrometry

**Methods**

**Synthetic matrix assisted laser desorption ionisation (MALDI) data**

**
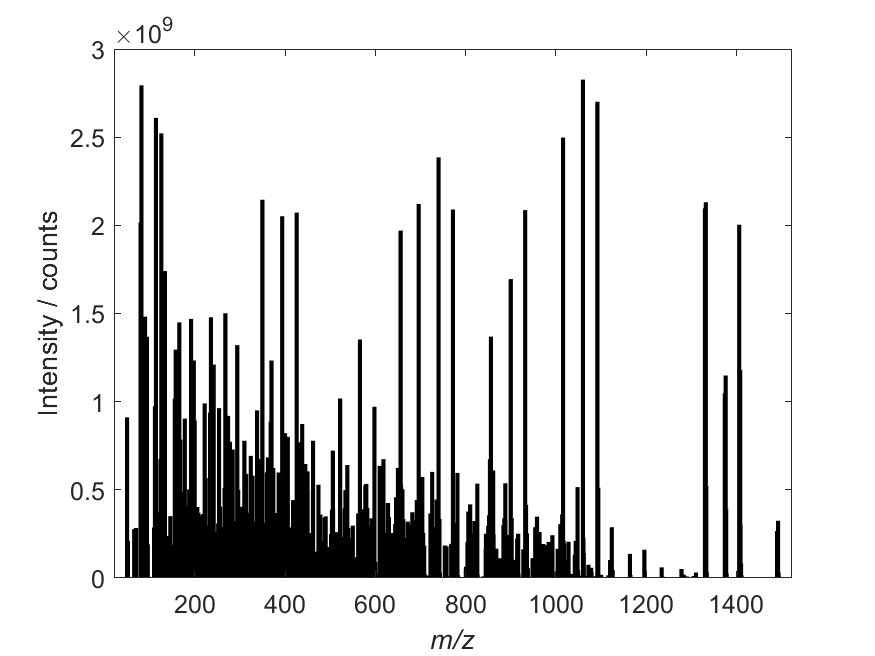
**

Figure 1 Example total mass spectrum of the synthetic MALDI dataset

**
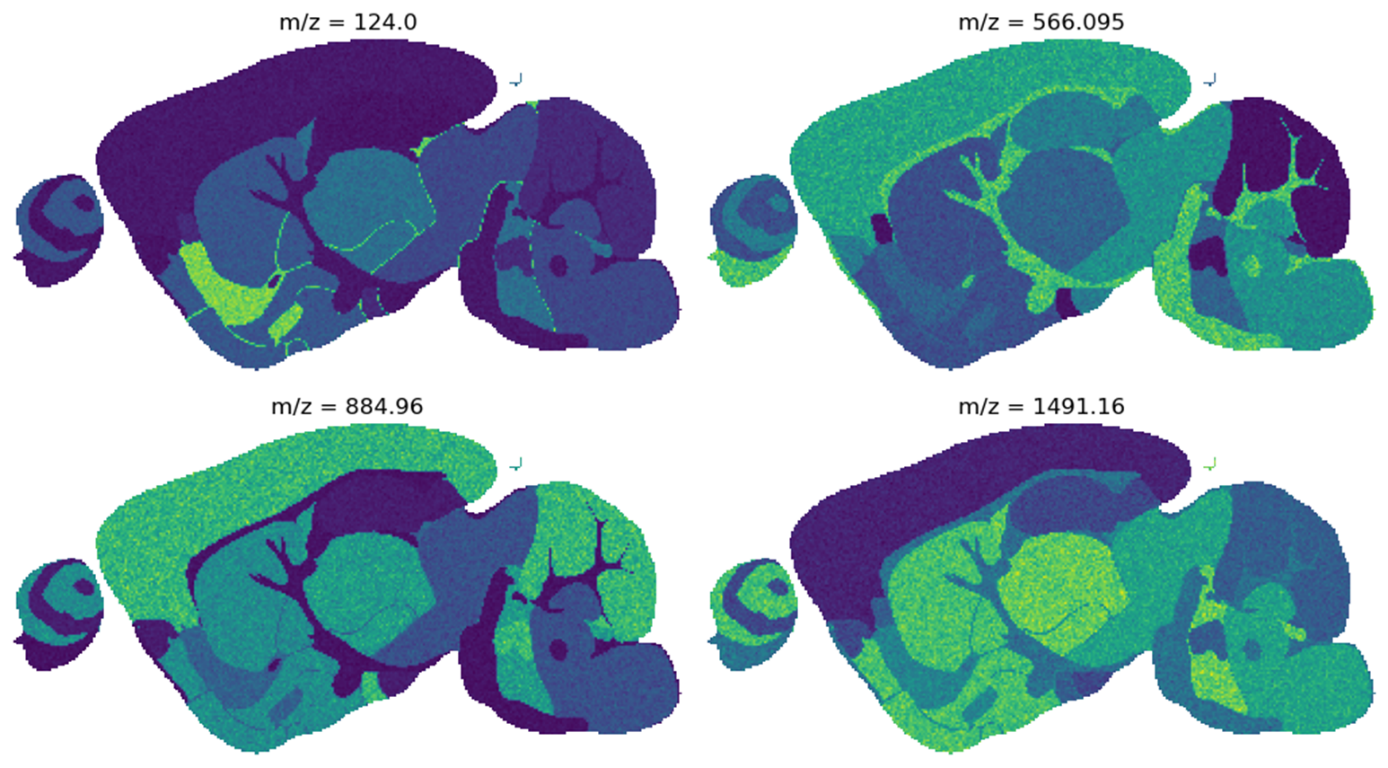
**

Figure 2 Four example single ion images for the synthetic MALDI dataset.

**t-SNE method**

| Dataset | Perplexity | Exaggeration |
| --- | --- | --- |
| synthetic MALDI | 1, 2, 3, 4, 5, 10, 20, 30, 40, 50, 100, 250, 500, 750, 1000, 1500, 3000, 5000, 7500, 10000 | 1, 2, 3, 4, 5, 10, 20, 30, 40, 50, 100, 250, 500, 750, 1000 |
| More homogeneous synthetic MALDI, 50 μm DESI sagittal brain, 50 μm MALDI transverse brain (no background) | 1, 4, 5, 10, 30, 50, 100, 250, 500, 750, 1000, 1500, 3000, 5000, 7500, 10000 | 1, 4, 5, 10, 30, 50, 100, 250, 500, 750, 1000 |
| disparate synthetic MALDI, synthetic MALDI + more homogeneous synthetic MALDI, DESI transverse brain | 1, 4, 5, 10, 30, 50, 100, 250, 500, 750, 1000, 1500, 3000, 5000, 7500 | 1, 4, 5, 10, 30, 50, 100, 250, 500, 750, 1000 |
| 20 μm MALDI sagittal brain (coarse) | 100, 250, 500, 1000, 1500, 3000 | 5, 10, 20, 50, 100, 250 |
| 20 μm MALDI sagittal brain (fine) | 1, 5, 10, 25, 50, 100, 250, 500, 1000, 1500, 2000, 2500, 3000 | 1, 5, 10, 25, 50, 100 |
| 50 μm MALDI transverse brain (background) | 1, 2, 3, 4, 5, 10, 20, 30, 40, 50, 100, 250, 500, 1000, 1500, 3000, 5000, 7500, 10000 | 1, 2, 3, 4, 5, 10, 20, 30, 40, 50, 100, 250, 500, 1000, 1500, 3000 |

Table 3 Hyper-parameter combinations for each dataset

**Cluster preservation evaluation**


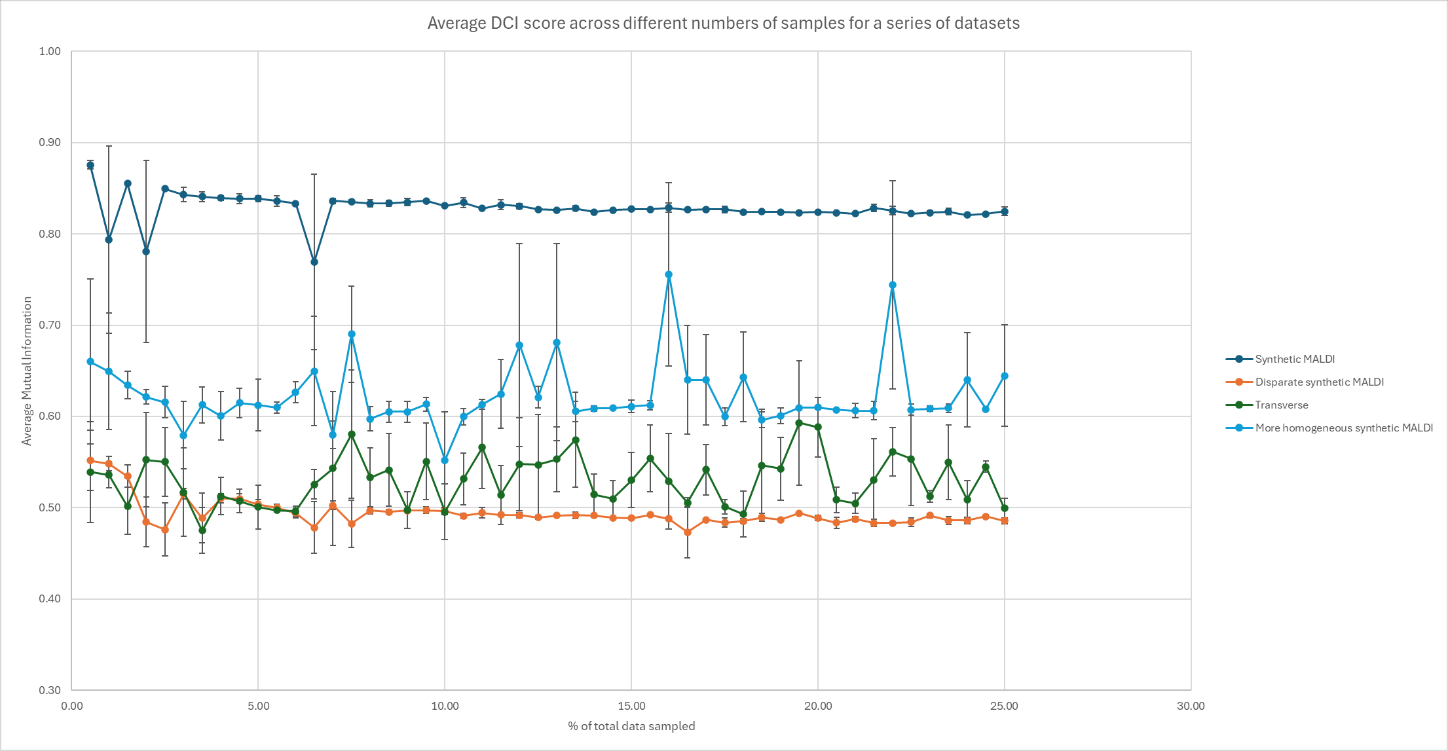


Figure 3 The average mutual information of 3 runs for a series of sample numbers as a % of total data samples for the Synthetic MALDI, disparate synthetic MALDI, more homogeneous synthetic MALDI, and Transverse datasets. The t-SNE hyperparamaters used in this were as follows; synthetic MALDI perplexity 7500, exaggeration 4, disparate synthetic data perplexity 7500 exaggeration, 500, transverse brain dataset perplexity 1500, exaggeration, 50, and more homogeneous dataset perplexity 5000, exaggeration 750.

1. Sampling of datapoints
   1. To generate a subset of the total data that are representative of a variety of the features present, kmeans clustering is applied to the low dimensional space with $k=\frac{n}{100}$ where n = total samples in the data. For further parameters, initialisation = random, number_initialisation = 10, max_iterations = 300, random_state = 42, all other parameters remained as default using the sklearn implementation.^17^ sklearn.cluster.KMeans function version 1.4. Typically, sampling involved with dimensionality reduction evaluation uses random sampling which can be done on pixels of high dimensional space, however in the making of this work it was found that sampling in this way does not guarantee the sampling/ identification of pixels misplaced in the embedding process of dimensionality reduction. Considering the poor embedding of a pixel is indicative of an insufficient embedding, it is important that these are considered in the evaluation method. The closest data points (Euclidean distance) to each cluster centroid were then taken to give a subset of the total data that is representative of the features present in the whole data. Pairwise distance for the low and high dimensional space were calculated using Euclidean and cosine distances respectively utilising the Scipy^18^ python library function scipy.spatial.distance.pdist version 1.13.1. Cosine distance was chosen as it is the metric used in the t-SNE embedding process
2. Hierarchical clustering of samples in high and low dimensions
   1. The condensed distance matrix for high and low dimensional space are used to generate a linkage matrix (average linkage) using the public library Scipy^18^, function scipy.cluster.hierarchy.linkage version 1.13.1 with cosine and Euclidean distance for high and low dimensional space respectively. The main benefit to hierarchical clustering in this way is that determination of cluster number is not necessary as each sample is iteratively merged into clusters.
3. Creation of cophenetic distance matrices based on the hierarchical clustering
   1. Cophenetic distance is a metric which measures the similarity between two data points based on their height (distance) in a hierarchical clustering dendrogram and these matrices were calculated from the linkage matrices using Scipy^18^ function scipy.cluster.hierarchy.cophenet version 1.13.1. Creation of the cophenetic distance matrix allows for the easy identification of sample relationships visually, in addition to allowing the user to inspect any differences between high and low dimensional space cophenetic distance relationships.
4. Mutual information calculation between high and low dimensional cophenetic distance matrices.
   1. The commonly used Pearson correlation is inappropriate for use with this method as it is sensitive to outliers, the cophenetic distances are not monotonic considering multiple samples can have the same distance, and this data is also not normally distributed. An arithmetic mean normalised mutual information, where mutual information is the shared information between variables, indicating how much knowledge of one reduces uncertainty of the other was calculated between condensed high and low dimensional cophenetic distance matrices using scikit-learn^17^ function sklearn.metrics.normalized_mutual_info_score version 1.4 with default parameters. Mutual information is utilised within this method because it is less sensitive to outliers, and considering there is a finite number of cophenetic distances a sample can have, this represents discrete data which is commonly used for MI. Mutual information does not assume any distribution of the data making it a flexible method in capturing linear, and non-linear relationships.

**Synthetic MALDI**


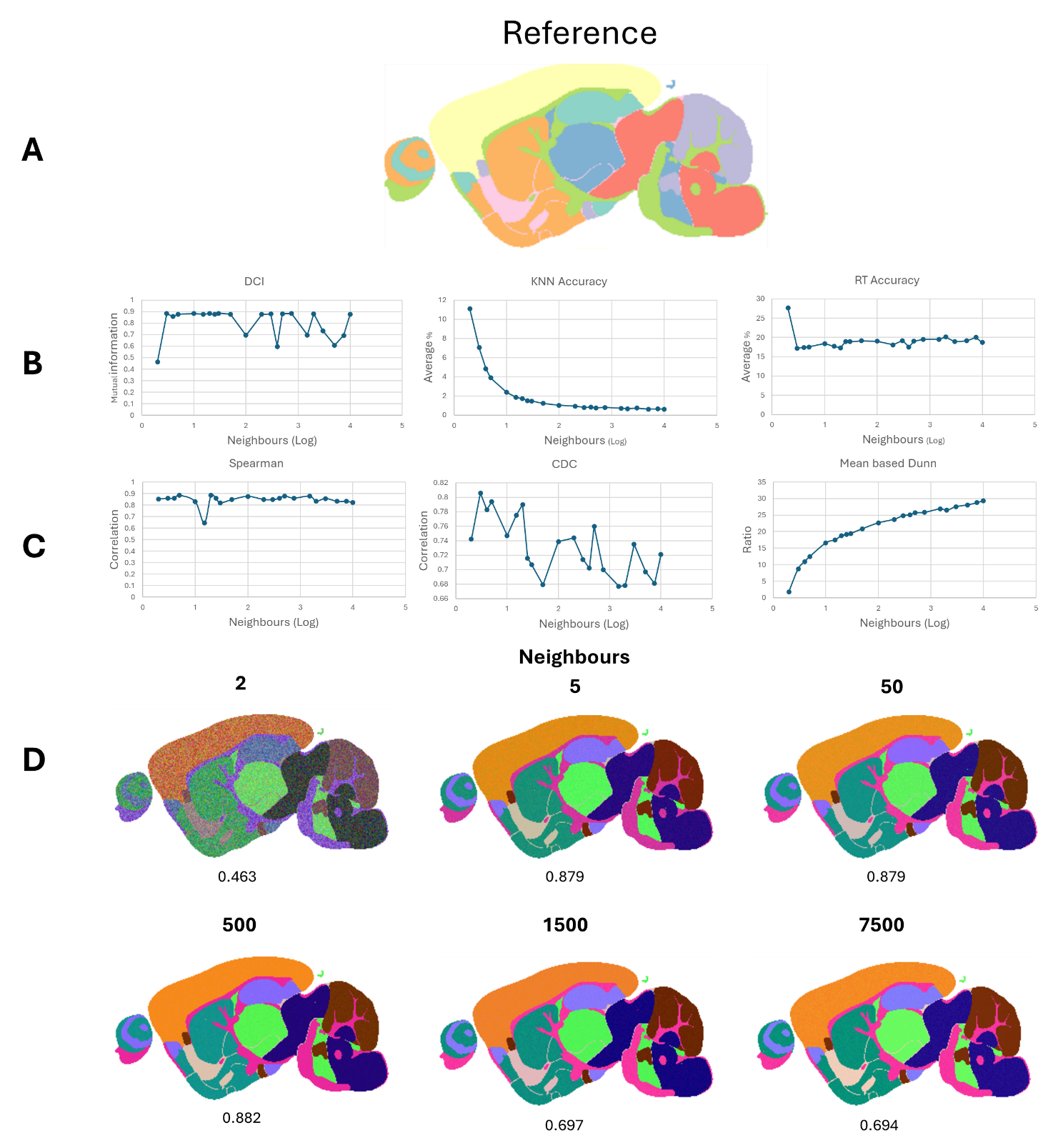


Figure 4 (A) Synthetic MALDI reference image. (B) Plot of DCI, KNN accuracy, and RT accuracy for a range of neighbours, respectively. (C) Plot of Spearman rank, Centroid distance correlation, and mean based Dunn for a range of neighbours, respectively. (D) UMAP 3D reduced representation embeddings of a range of neighbours with their associated DCI results.


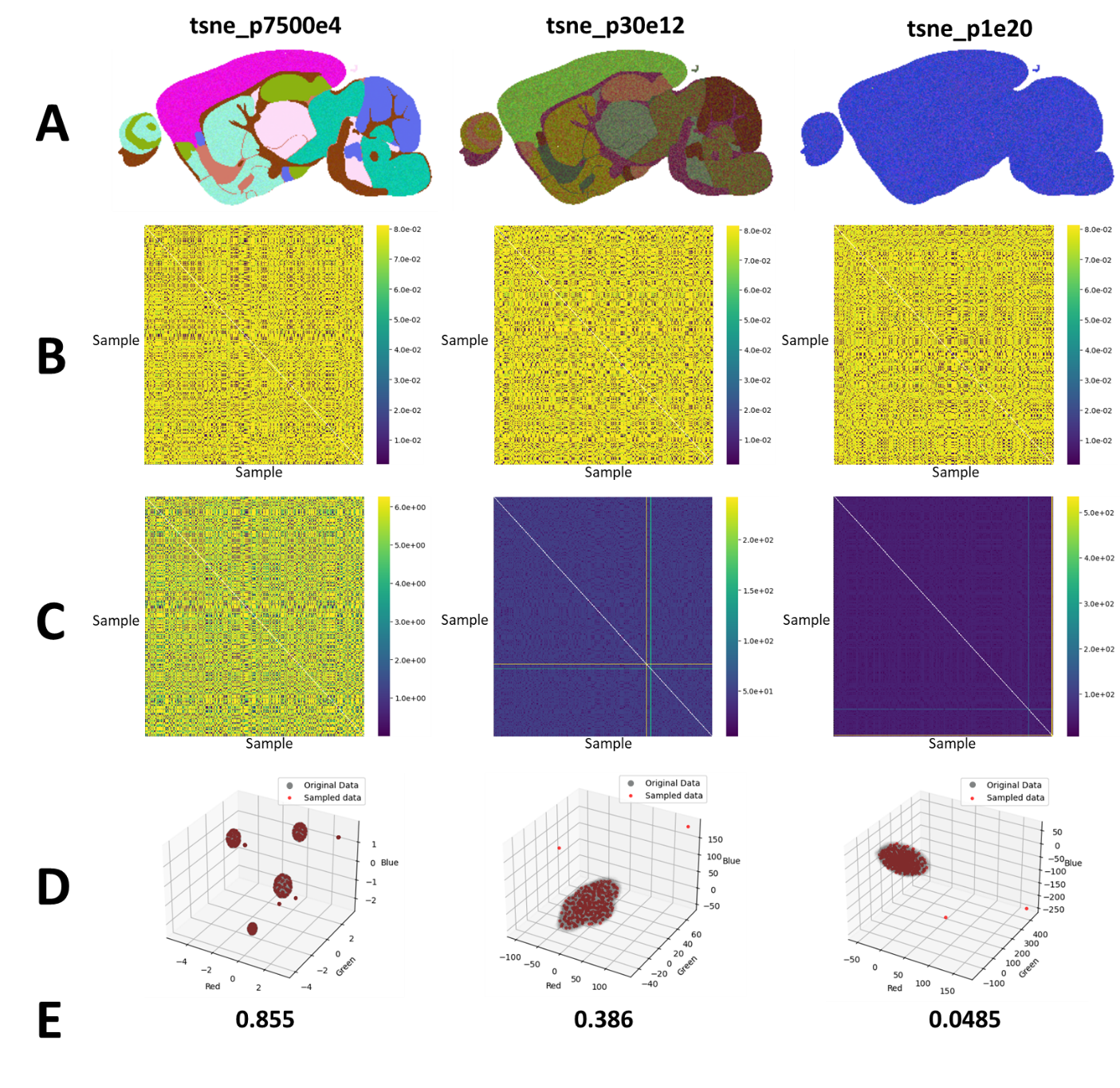


Figure 5 (A) Embeddings for different combinations of t-SNE hyper-parameters. (B) The pairwise cophenetic distance matrix between sampled points for the high dimensional space. (C) The pairwise cophenetic distance matrix between sampled points for the low dimensional space. (D) A 3D scatter plot of the t-SNE embeddings, including labelling of sampled data points. (E) The mutual information between the high dimensional pairwise cophenetic distance matrix, and the t-SNE embedding pairwise cophenetic distance matrix.

A sample of t-SNE reduced representations of the synthetic MALDI dataset are shown in Figure 5, where *perplexity 7500, exaggeration 4* (p7500e4) is the embedding with one of the greatest mutual information scores (0.855), *perplexity 30 exaggeration 12* is an embedding with a “medium” mutual information (0.386), and *perplexity 1 exaggeration 20* is representative of embeddings with a mutual information of approximately 0. This figure shows how the visual similarity between the 3D embedding and the reference image changes over different values of mutual information, and it can be seen that the scores are indicative of visual similarity.

Figure 5, row B also shows that the similarity between the pairwise cophenetic distance matrix between the high dimensionally sampled data points and t-SNE embedding sampled data points increases with larger mutual information scores. The similarity between these matrices is also indicative of visual similarity. Not only the two low quality embeddings show poor overall cluster preservation in the cophenetic distance matrices, but also show that some samples are completely misplaced in lower dimensional space.

The 3D scatter plots in row C highlights the distribution of data points within each embedding, as well as visualising which data points have been sampled (red). For the *perplexity 7500 exaggeration 4* embedding, the distribution of data points is well defined, with clear and compact clusters within the data. The data points in the 3D scatter for *perplexity 30 exaggeration 12* are more diffuse, with less distinct clusters, therefore is less appropriate than *perplexity 7500 exaggeration 4* which more closely resembles the true underlying patterns of the original high dimensional data. *Perplexity 1 exaggeration 20* has inappropriately embedded many pixels, leading to an inappropriate reduced representation with some data points that do not belong to any similar cluster, which the mutual information score reflects.


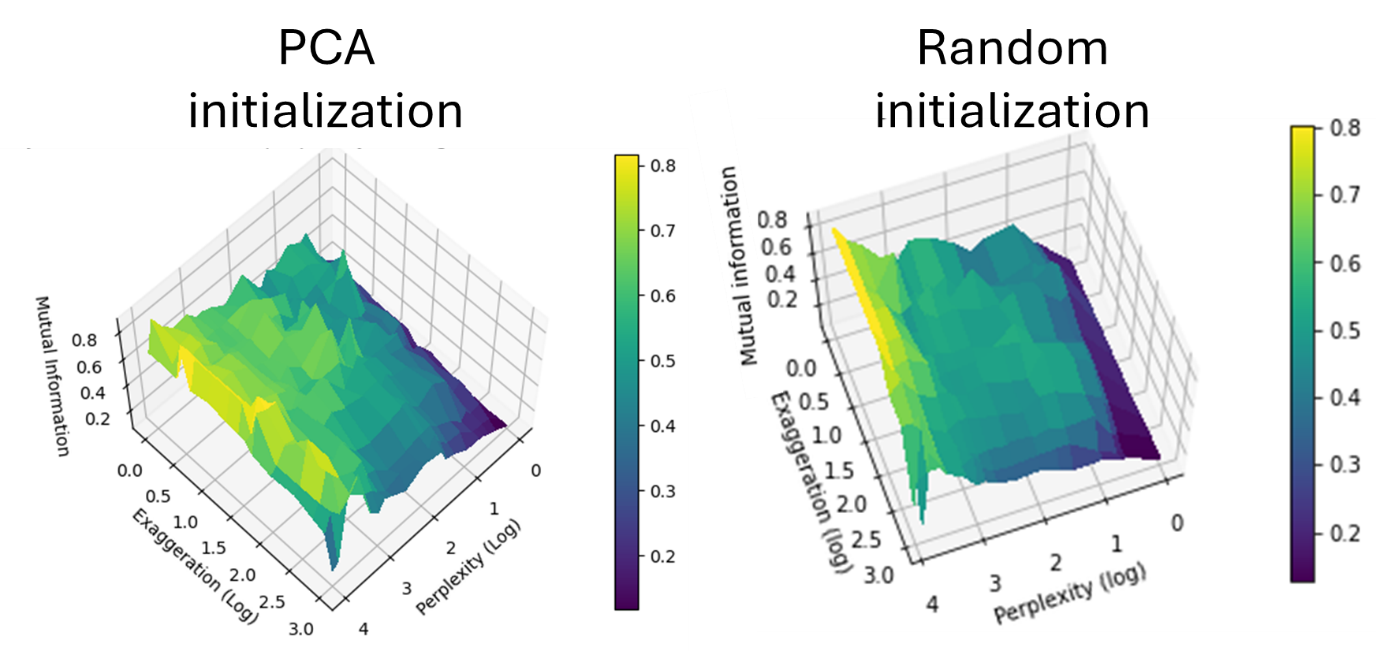


Figure 6 Synthetic MALDI DCI scores for a range of perplexity and exaggeration values with PCA initialisation (left) and a random initialisation (right)

**Disparate synthetic dataset**


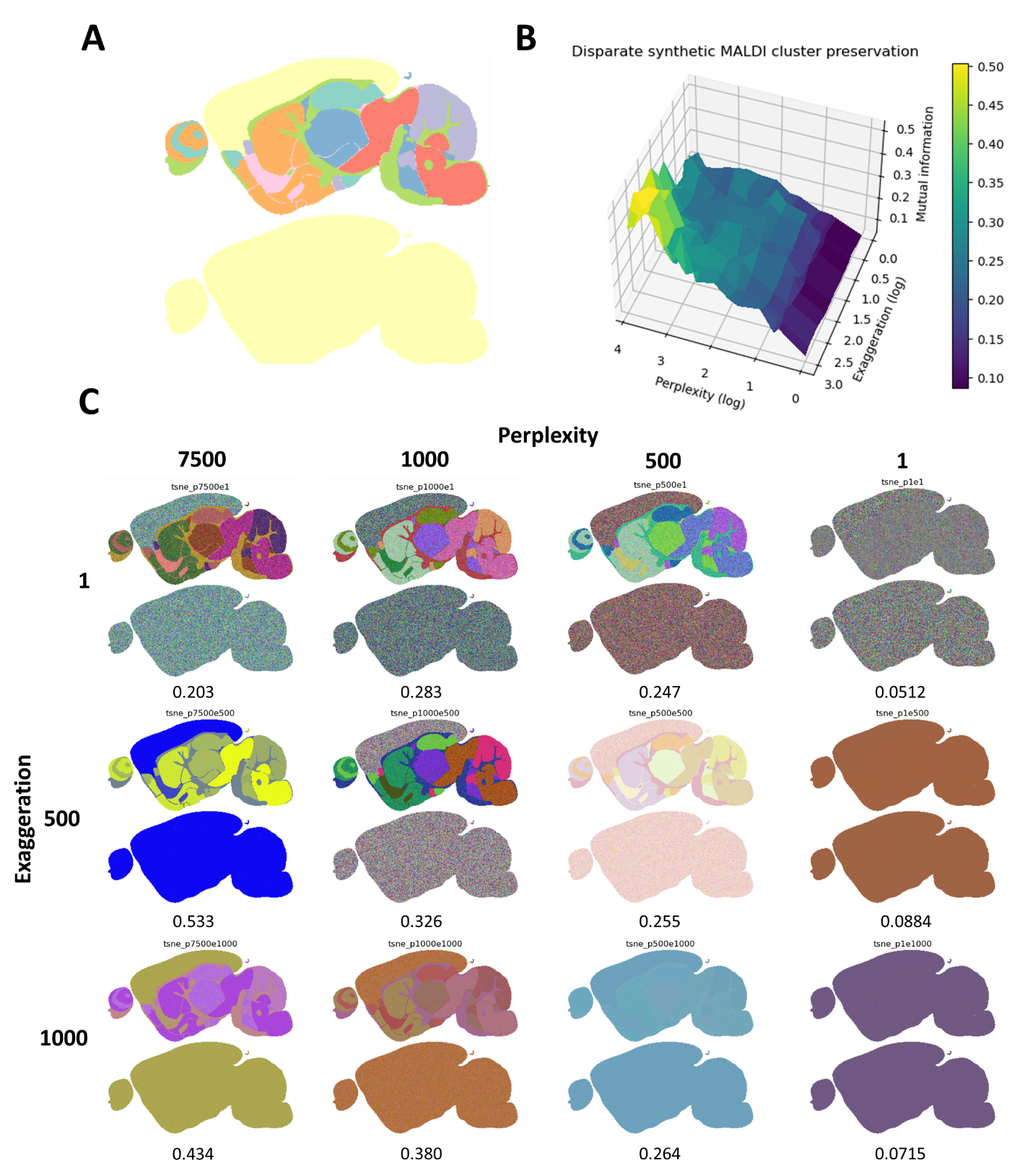


Figure 7 (A) Reference image of the Synthetic MALDI + disparate cluster dataset. (B) Grid plot of the t-SNE hyper parameters perplexity vs exaggeration, and their associated cophenetic distance matrix mutual information when compared with the high dimensional cophenetic distance matrix. (C) 3D t-SNE embeddings of the Synthetic MALDI + disparate cluster dataset, perplexity values along the top, exaggeration along the left.

The crowding problem is a phenomenon caused by intrinsically highly dimensional datasets being embedded in a smaller dimension, and this could be exacerbated with the inclusion of the disparate cluster. The clusters are overlapping with each other due to the reduced space for the embedded features. The disparately large cluster, as well as the cluster it was sampled from also appear to be incredibly “noisy”. This might be due to differences in data density within this cluster, that t-SNE tries to separate leading to a less homogenate cluster.

These hypotheses may also explain why larger values of perplexity lead to higher mutual information scores for this dataset; larger values of perplexity lead to more neighbours being considered, and thus more global feature preservation which is more important for this dataset. An exaggeration of 250 or larger existed for the top 10 highest scoring hyperparameter combinations; a larger value of exaggeration may lead to better cluster preservations for this dataset since it helped to distinguish clusters from each other which is more crucial with the larger cluster present.


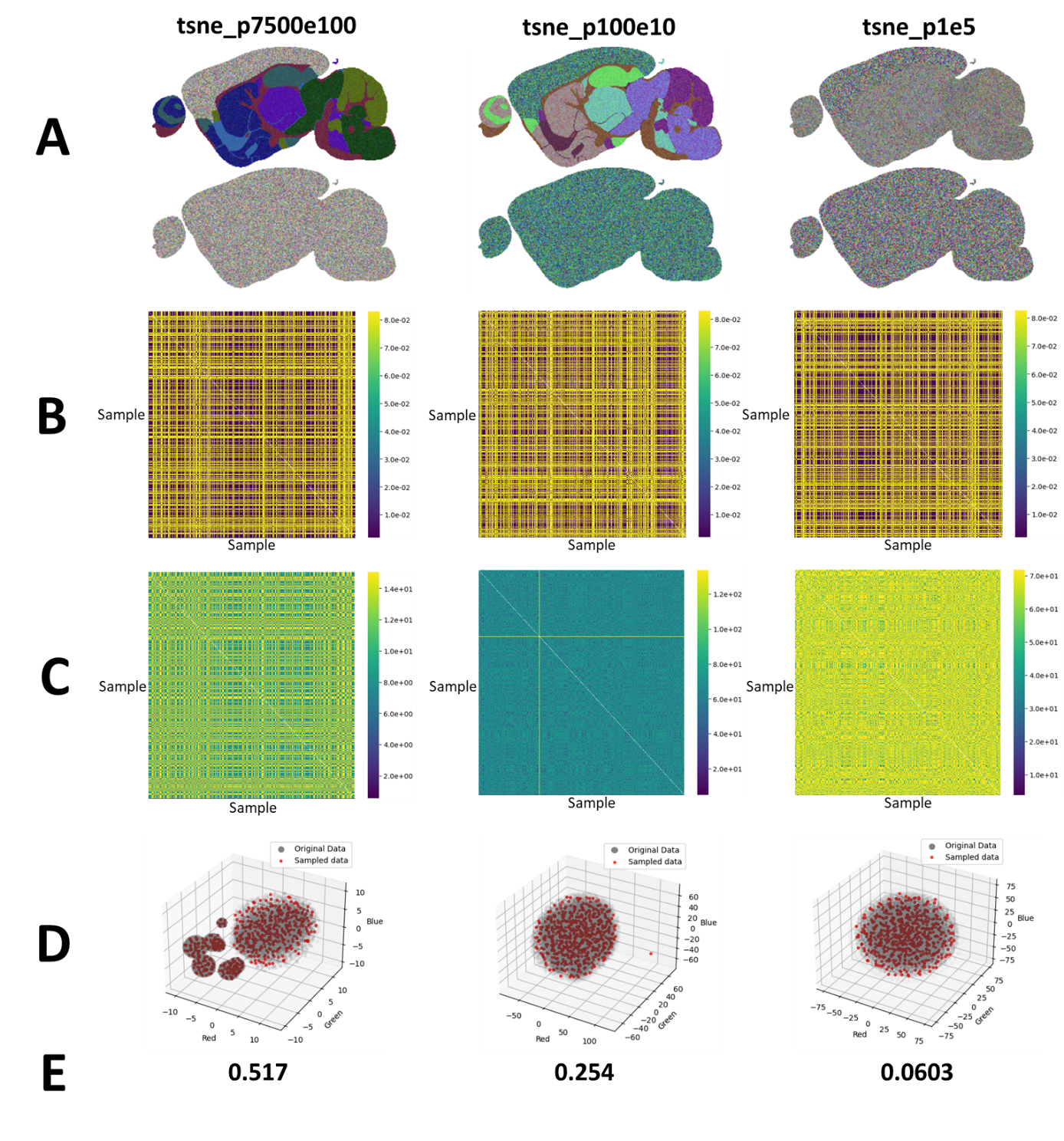


Figure 8 (A) Embeddings for different combinations of t-SNE hyper-parameters. (B) The pairwise cophenetic distance matrix between sampled points for the high dimensional space. (C) The pairwise cophenetic distance matrix between sampled points for the low dimensional space. (D) A 3D scatter plot of the t-SNE embeddings, including labelling of sampled data points. (E) The mutual information between the high dimensional pairwise cophenetic distance matrix, and the t-SNE embedding pairwise cophenetic distance matrix.

Figure 8 demonstrates the t-SNE reduced representations of the synthetic MALDI + disparate cluster dataset, where *perplexity 7500 exaggeration 100* is the embedding with the greatest mutual information, *perplexity 100 exaggeration 10* is an embedding with a “medium” mutual information, and *perplexity 1 exaggeration 5* is representative of embeddings with a mutual information of approximately 0. It can also be seen how the visual similarity between the 3D embedding and the reference image changes over different values of mutual information.

Not only are the mutual information scores much lower for this dataset, it can be seen that the visual similarity between the cophenetic distance matrices to the reference is also much lower. Some similarity between the cophenetic distance matrices between the reference and p7500e100 can be seen, however it is not clear. This dissimilarity is also demonstrated by the 3D scatter plot where some clusters appear distinct, however most data points remain within a diffuse cluster in the centre.

The embedding image of *perplexity 100 exaggeration 10* shows clusters separated, however has a low score of 0.254. This illustrates the fact that an embedded representation of data may visually look satisfactory, whilst still having a low cluster preservation. The 3D scatter shows the clusters of data as very diffuse, with almost no discernible difference between them which matches the low mutual information. This could be a result of the low exaggeration. The *perplexity 1 exaggeration 5* embedding is visibly noisy, with no visibly correlating patterns between cophenetic distance matrices between itself and the reference, with a 3D scatter comprised of a single completely diffuse cluster without any separation indicative of its mutual information.


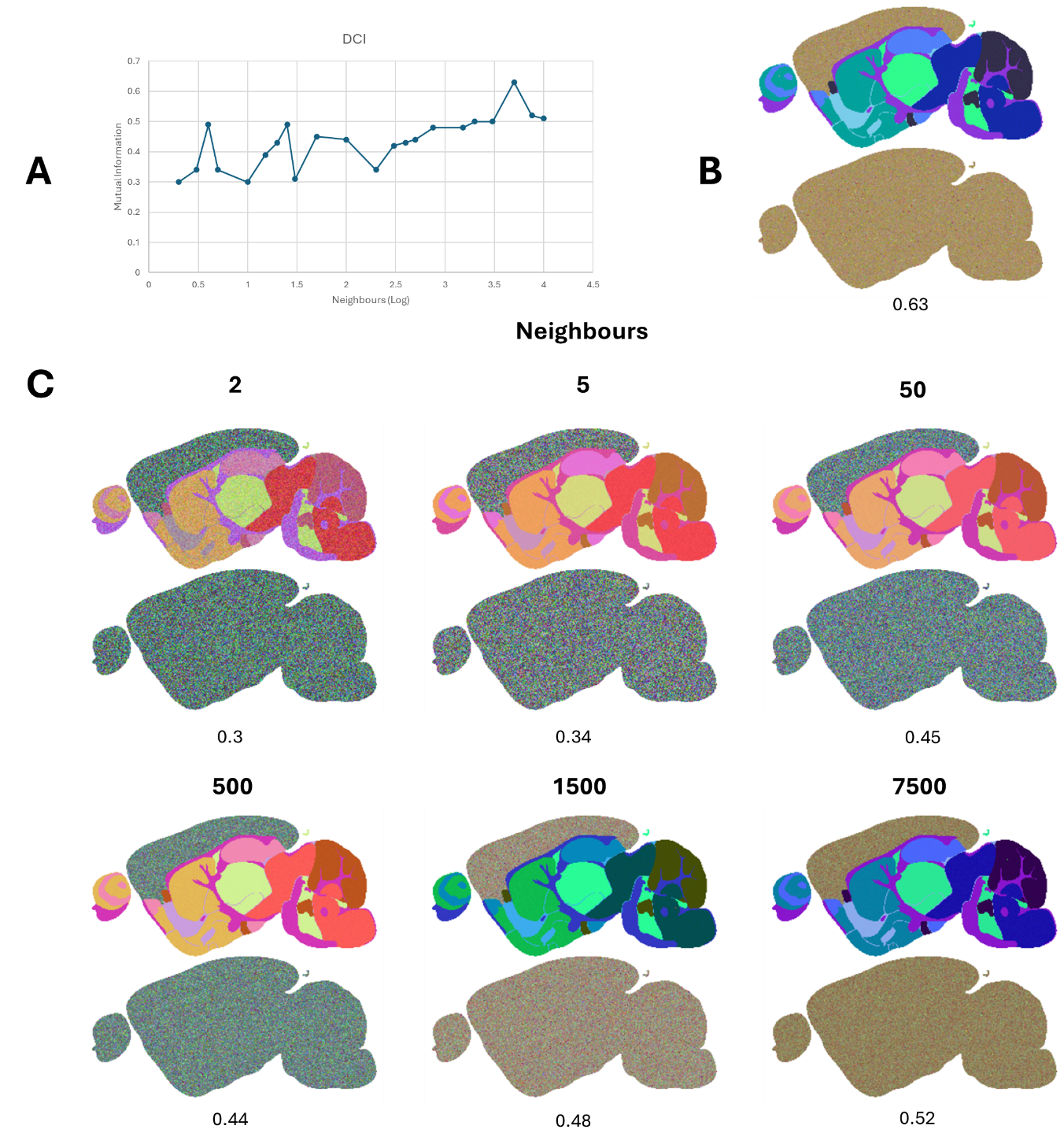


Figure 9 (A) Plot of DCI when performing UMAP on the disparate cluster size synthetic data for a range of neighbours. (B) UMAP embedding with the greatest score according to DCI using 5000 neighbours. (C) UMAP 3D reduced representation embeddings of a range of neighbours with their associated DCI results.

**More homogeneous synthetic MALDI**

An overview of mutual information scores for different combinations of hyper-parameters can be seen in Figure 10.


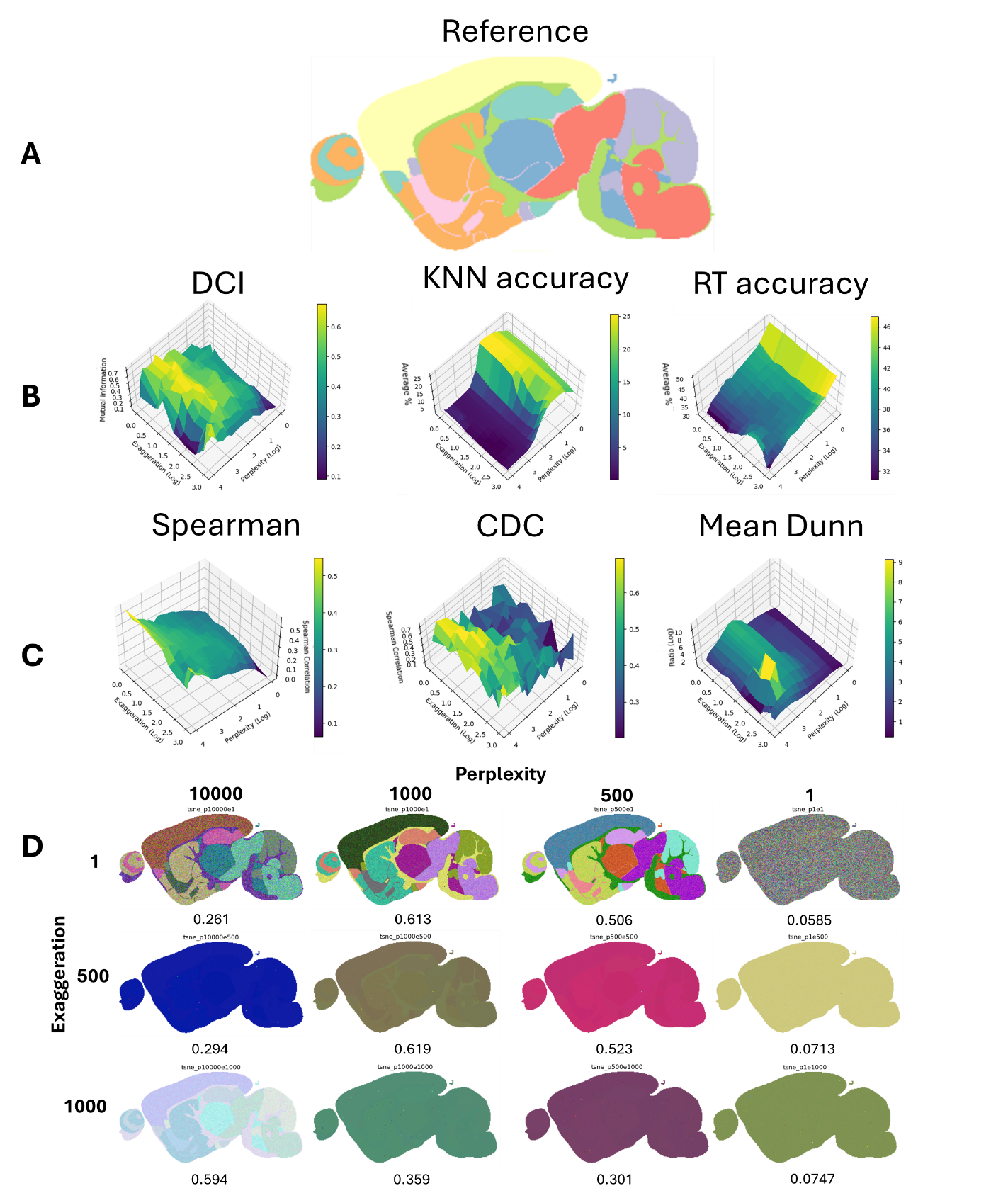


Figure 10 (A) Synthetic MALDI reference image. (B) Gridplot of DCI, KNN accuracy, and RT accuracy for a range of perplexity and exaggeration results, respectively. (C) Gridplot of Spearman rank, Centroid distance correlation, and mean based Dunn for a range of perplexity and exaggeration results, respectively. (D) 3D reduced representation embeddings of a range of Perplexity (left to right) and Exaggeration (top to bottom) with their associated DCI results.

The relationship between hyperparameter combination and mutual information is almost the same as that as for the synthetic MALDI dataset, however with overall lower scores. These lower scores are a result of the relationships between clusters being much more similar, and thus t-SNE doesn’t preserve these relationships as well as the distinct cluster case.


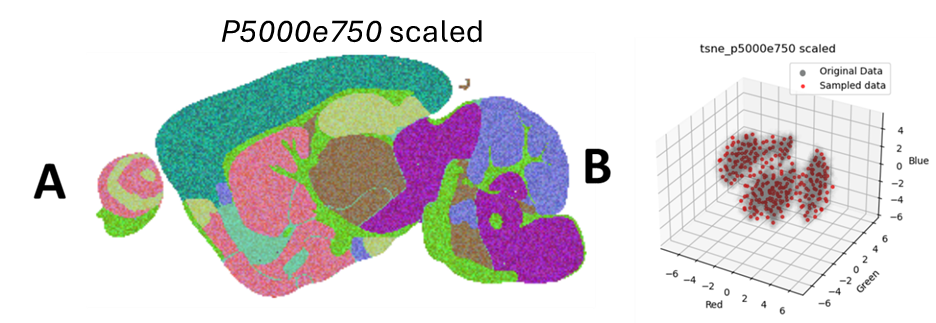


Figure 11 (A) p5000e750 embedding thresholded between the 0.01 and 99.9 percentile. (B) 3D scatter plot of the embedding.

Figure 11 shows the *p5000e750* embedding when thresholded between the 0.01 – 99.9 percentile. The groups of data within the embedding are very distinct from each other, with the 3D scatter also showing distinct clusters within this dataset.


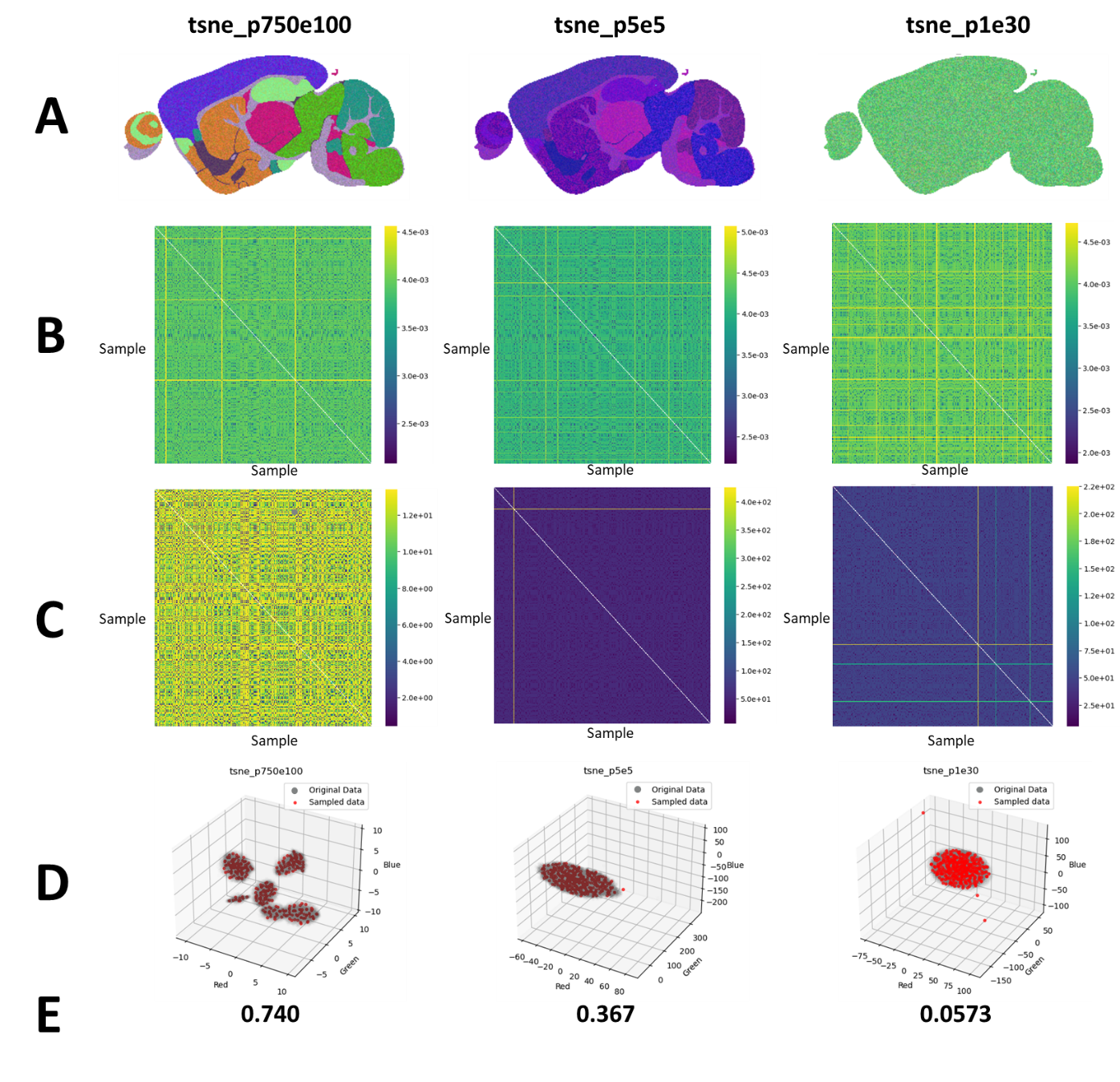


Figure 12 (A) Embeddings for different combinations of t-SNE hyper-parameters. (B) The pairwise cophenetic distance matrix between sampled points for the high dimensional space. (C) The pairwise cophenetic distance matrix between sampled points for the low dimensional space. (D) A 3D scatter plot of the t-SNE embeddings, including labelling of sampled data points. (E) The mutual information between the high dimensional pairwise cophenetic distance matrix, and the t-SNE embedding pairwise cophenetic distance matrix.

Figure 12 shows 3 different t-SNE embeddings of the more homogeneous synthetic MALDI data, along with their respective scores. *Perplexity 750 exaggeration 100* is the embedding with one of the greatest mutual information scores, *perplexity 5 exaggeration 5* represents embeddings of ~0.35 mutual information, and p1e30 is representative of embeddings with scores of ~0. As with the synthetic MALDI dataset, the mutual information are indicative of the visual similarity between the reference image and the embedding images.

Upon close inspection of the cophenetic distance matrices in row C of figure 12, it can be seen that only the matrix of *perplexity 750 exaggeration 100* resembles that of the reference. In order to ascertain the reason for lower scores compared to the synthetic MALDI data, and to understand if the relationships between distant or close datapoints are being preserved differently, these cophenetic distance matrices can be inspected. Comparing the matrix produced by *perplexity 750 exaggeration 100* to the reference matrix, shows that the cophenetic distance matrix for the embedding has exaggerated the relationships between pixels. There are not many “middle distance” or “mid-near” pixel relationships in the embedding matrix, however appears to be the dominant relationship in the reference matrix. Distant sample relationships shown in the reference matrix are not as clearly distinguishable in the *perplexity 750 exaggeration 100* embedding matrix.
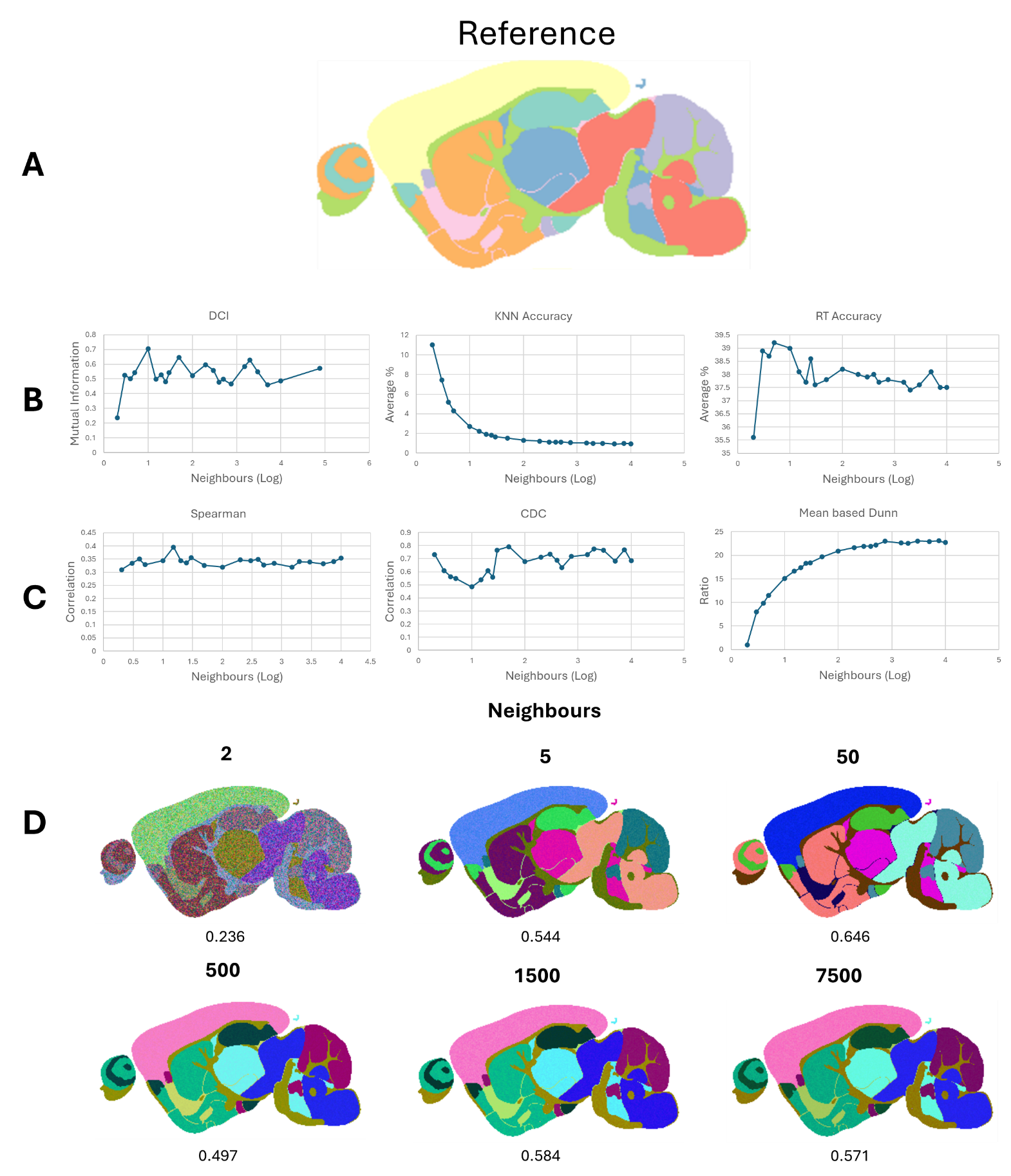


Figure 13 (A) Synthetic MALDI reference image. (B) Plot of DCI, KNN accuracy, and RT accuracy for a range of neighbours, respectively. (C) Plot of Spearman rank, Centroid distance correlation, and Inter/Intra distance ratio for a range of neighbours, respectively. (D) UMAP 3D reduced representation embeddings of a range of neighbours with their associated DCI results.

**Synthetic MALDI + more homogeneous synthetic MALDI**


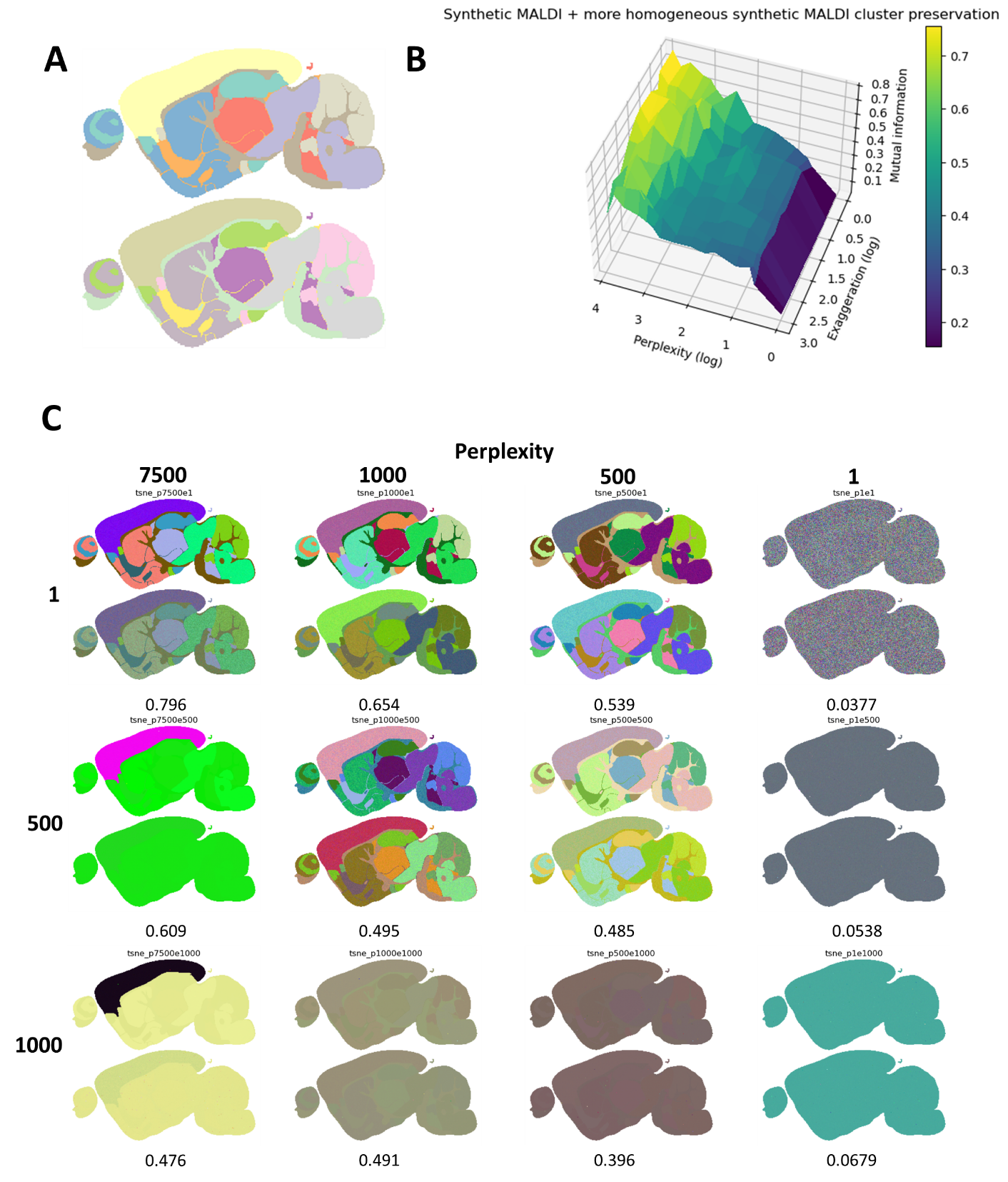


Figure 14 (A) Reference image of the synthetic MALDI + more homogeneous synthetic MALDI dataset. (B) Grid plot of the t-SNE hyper parameters perplexity vs exaggeration, and their associated cophenetic distance matrix mutual information when compared with the high dimensional cophenetic distance matrix. (C) 3D t-SNE embeddings of the synthetic MALDI + more homogeneous synthetic MALDI dataset, perplexity values along the top, exaggeration along the left.

The overview of results shown in Figure 14 are similar to that of the synthetic MALDI dataset, and more homogeneous synthetic MALDI dataset which makes sense considering their fundamental similarity.


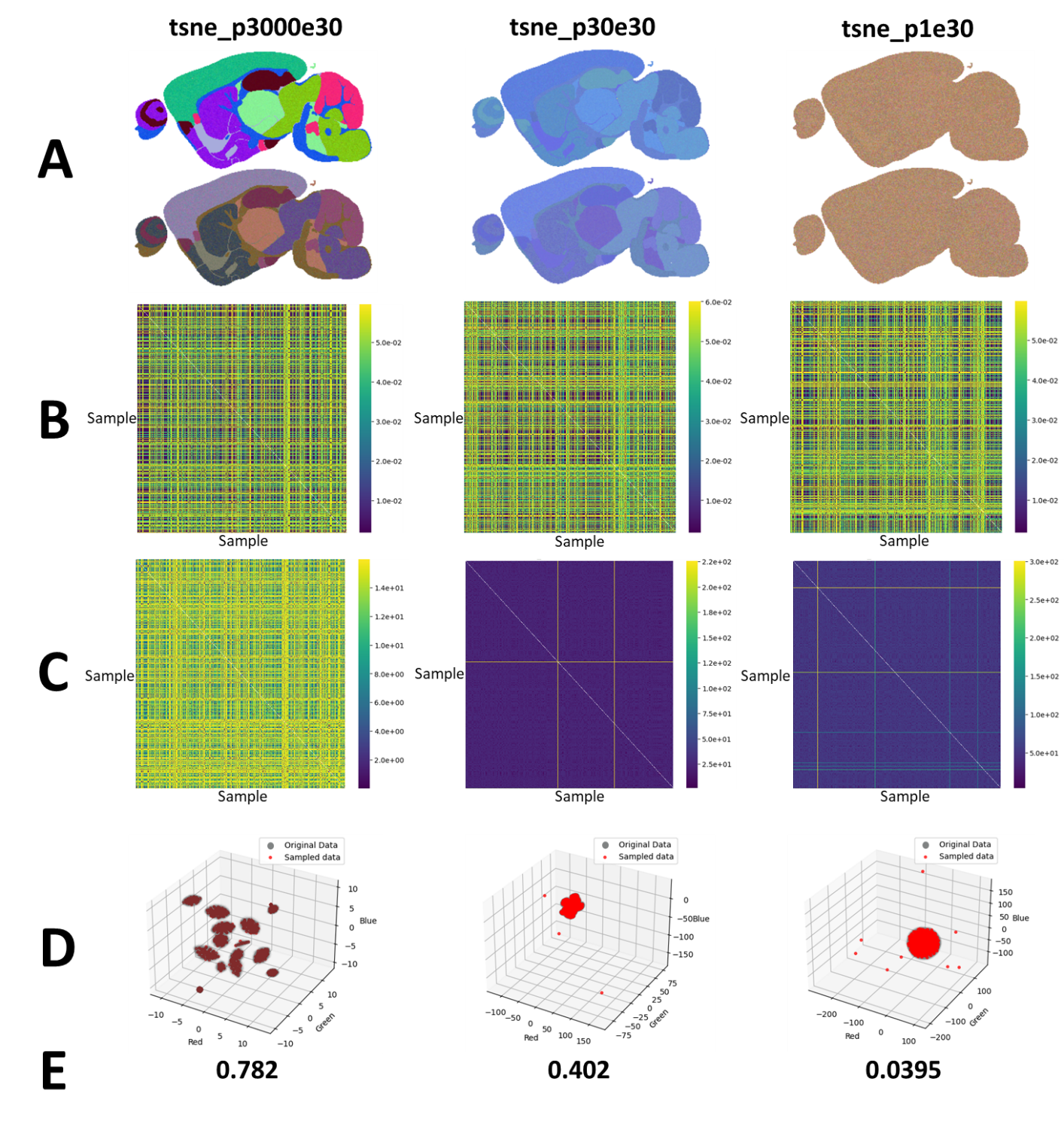


Figure 15 (A) Embeddings for different combinations of t-SNE hyper-parameters. (B) The pairwise cophenetic distance matrix between sampled points for the high dimensional space. (C) The pairwise cophenetic distance matrix between sampled points for the low dimensional space. (D) A 3D scatter plot of the t-SNE embeddings, including labelling of sampled data points. (E) The mutual information between the high dimensional pairwise cophenetic distance matrix, and the t-SNE embedding pairwise cophenetic distance matrix.

Figure 15 shows three different embeddings across a range of mutual information scores. The cophenetic distance matrix of *perplexity 3000 exaggeration 30* shows similarity to that of the reference, however with an obvious difference; sampled pixels which have low cophenetic distance (and therefore closely related) when looking at the reference matrix, are not as closely related when looking at the matrix for *perplexity 3000 exaggeration 30*. There is not as distinct a difference between samples which are closely related and samples which are distant from each other.

**DESI sagittal brain**


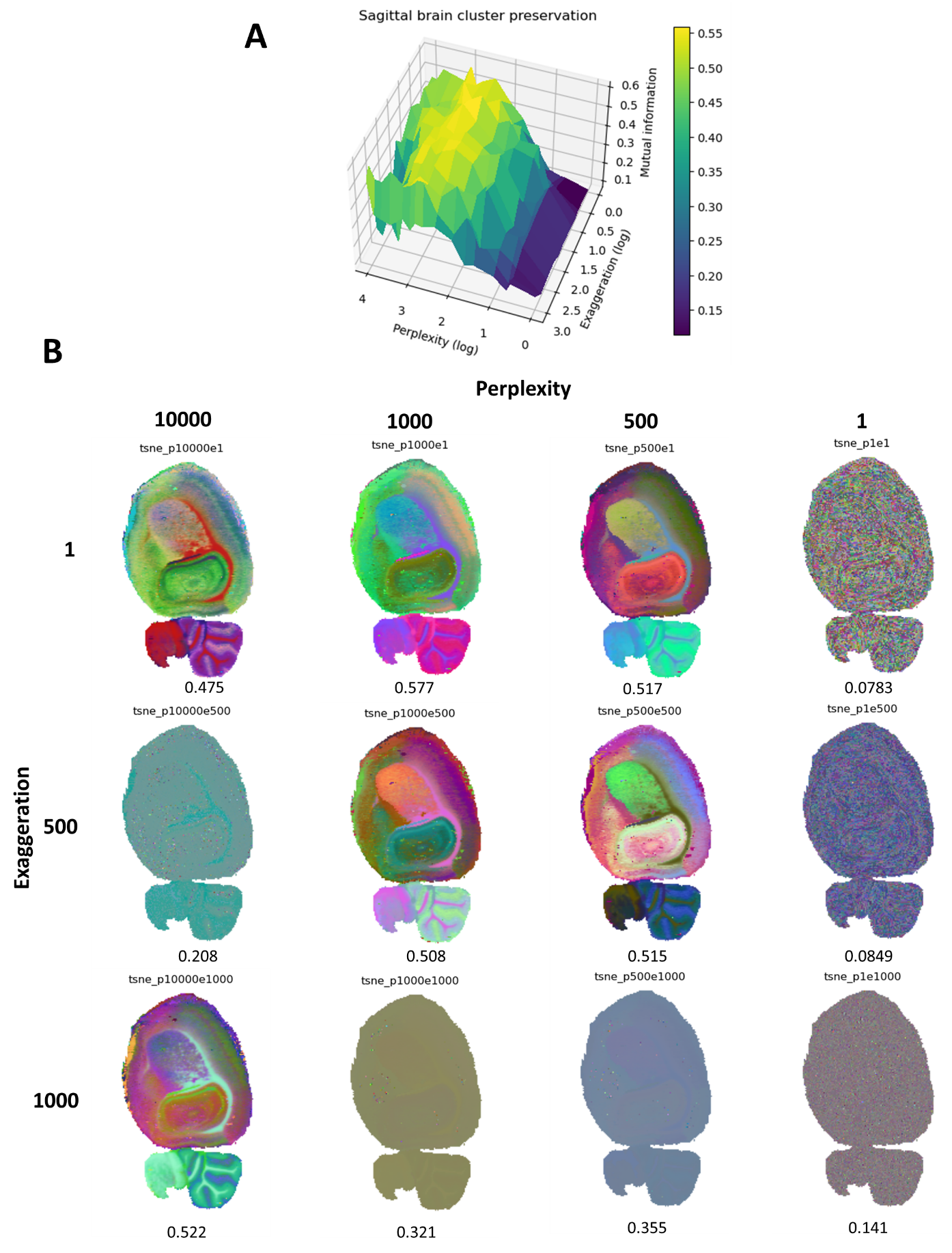


Figure 16 (A) Grid plot of the t-SNE hyper parameters perplexity vs exaggeration, and their associated cophenetic distance matrix mutual information when compared with the high dimensional cophenetic distance matrix. (B) 3D t-SNE embeddings of the sagittal brain dataset, perplexity values along the top, exaggeration along the left.

Figure 16 shows a similar relationship between mutual information and hyperparameter combination that has been shown for the majority of synthetic MALDI datasets. Higher perplexity, and lower exaggeration leads to the greatest preservation of cluster relationships. There appears to be a peak in mutual information at ~log(3) perplexity, corresponding to the approximate perplexity range of 500-1500, where values of perplexity greater than this range tend toward a decrease. Exaggeration also does not seem to have as great an impact on mutual information score than perplexity does.


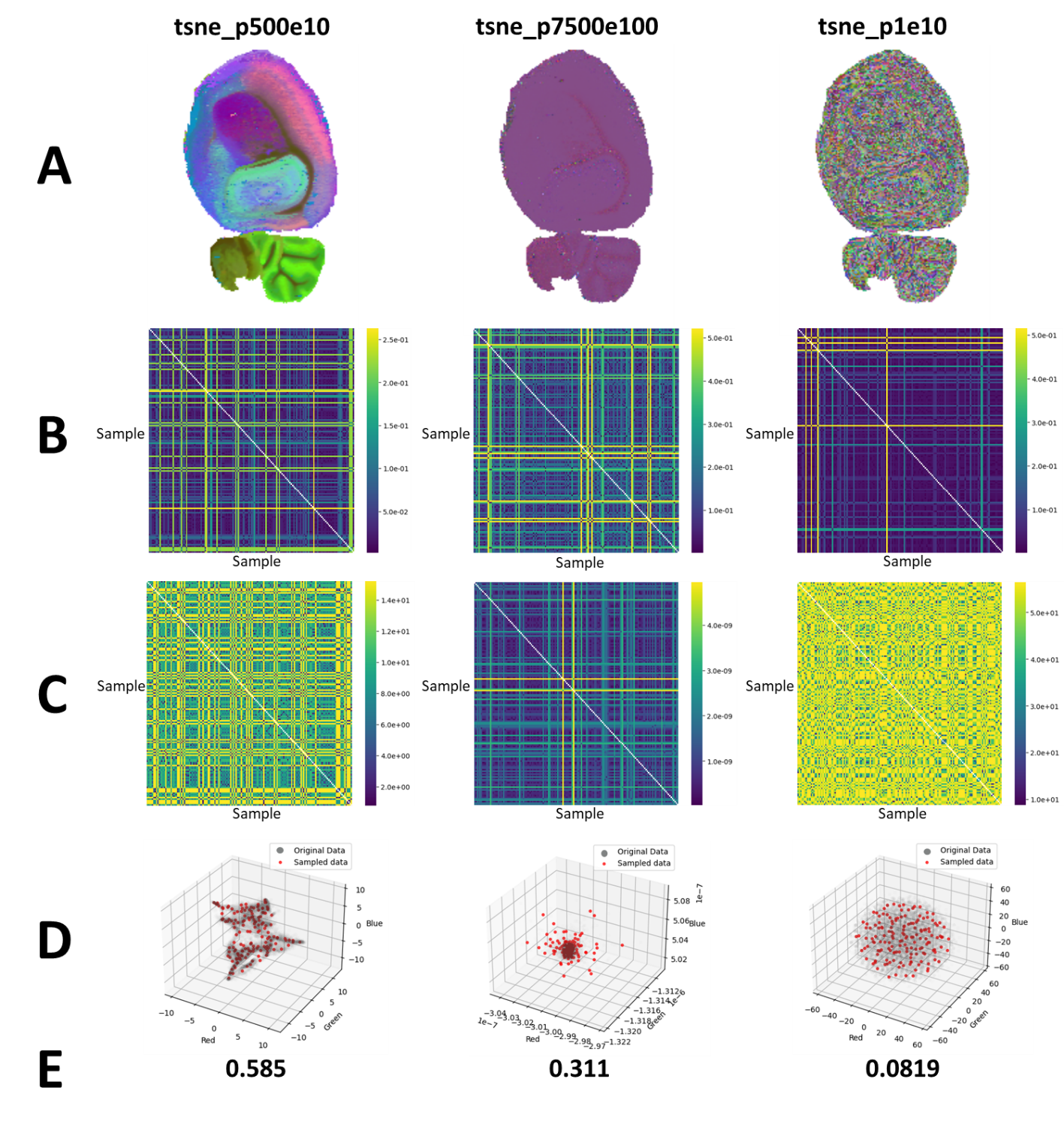


Figure 17 (A) Embeddings for different combinations of t-SNE hyper-parameters. (B) The pairwise cophenetic distance matrix between sampled points for the high dimensional space. (C) The pairwise cophenetic distance matrix between sampled points for the low dimensional space. (D) A 3D scatter plot of the t-SNE embeddings, including labelling of sampled data points. (E) The mutual information between the high dimensional pairwise cophenetic distance matrix, and the t-SNE embedding pairwise cophenetic distance matrix.

Figure 17 shows embeddings for a range of mutual information scores where perplexity 500 exaggeration 10 represents one of the embeddings with the greatest mutual information. There is no reference to compare these embeddings to, however the results gathered with the synthetic datasets provide some confidence in DCI’s ability to assess how well sampled data points relationships are maintained across dimensionality. The perplexity 7500 exaggeration 100 embedding looks visually poor with a low mutual information score of 0.311 where thresholding does not affect the visual quality of this embedding and perplexity 1 exaggeration 10 as with other datasets with such low perplexity values and similar mutual information score, looks visibly noisy.


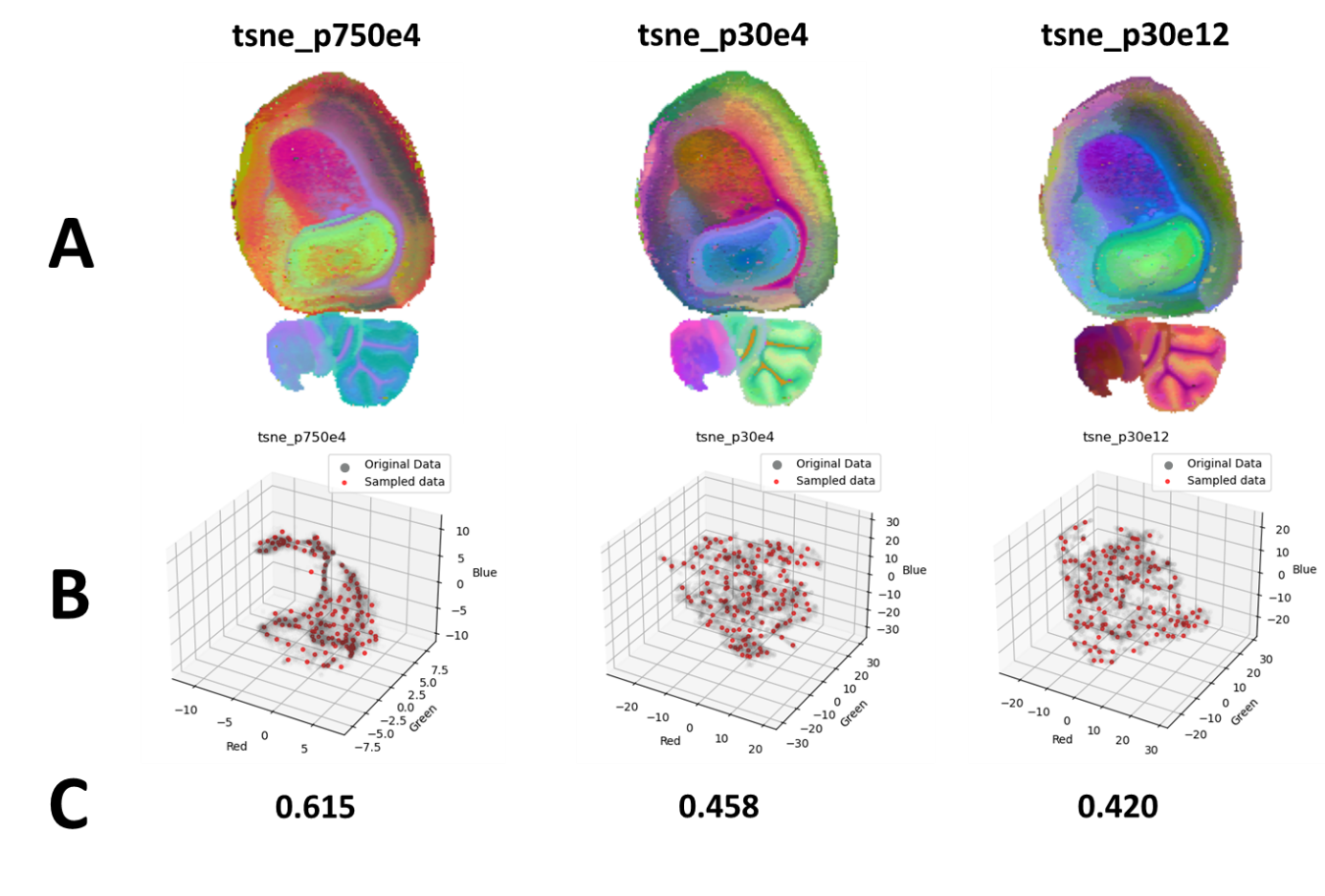


Figure 18 (A) Greatest embedding found by DCI, van der Maaten recommended hyper parameters, and Sklearn default hyper parameters. (B) 3D scatter plots for each embedding. (C) Mutual information scores between high and low dimensional space cophenetic distance matrices.

Figure 18 shows the highest quality embedding produced by DCI as well as the van der Maaten and Sklearn default hyper-parameter produced embeddings. Here, each embedding looks visually very similar, and a user may not think of any as better in preserving relationships within data than the other. The *perplexity 750 exaggeration 4* and *perplexity 30 exaggeration 4* embeddings appear to have clearer separation of layers within the cerebral cortex than *perplexity 30 exaggeration 12*, but it is difficult to discern any other difference in anatomical region separation. For this dataset, most of the best embeddings had perplexities between ~500 -1500 with a decrease in cluster preservation past these values, which could be due to local features and finer differences between clusters being more important in preserving cluster relationships.

Large values of perplexity have consistently yielded the highest quality embedding using DCI, when default and recommended perplexity ranges between 5-50. The original experiments using t-SNE by van der Maaten involved datasets typically much smaller than those of MSI datasets, for example, the frequently used MNIST dataset contains 60,000 samples and ~800 dimensions. The scale of MSI datasets exceed that of which t-SNE was originally designed for in addition to its inability to preserve global relationships. It is for this reason that higher values of perplexity may help to compensate for global feature preservation and becomes more appropriate for MSI datasets.

**20um MALDI Sagittal brain**

The coarse grid search is produced from 36 embeddings, with a total time of analysis of ~107 h. The time taken to produce these embeddings raise with the value of perplexity used where perplexity 100 embeddings required only 1 hour to produce, whereas perplexity 3000 embeddings required over 6 hours to produce. A grid search on the scale of the original synthetic MALDI dataset for this 20 μm MALDI sagittal brain dataset would take approximately 800 h (based on average times taken to produce embeddings with different hyper-parameter combinations for the 20 μm brain dataset). This is a time of an entire month, uninterrupted which is too time consuming for most studies. In addition to the large time of analysis required, the computational resources required are also great, and many users of this algorithm may not have access to the required computational power. It is already known that t-SNE is not very scalable with larger datasets and thus may not be appropriate.


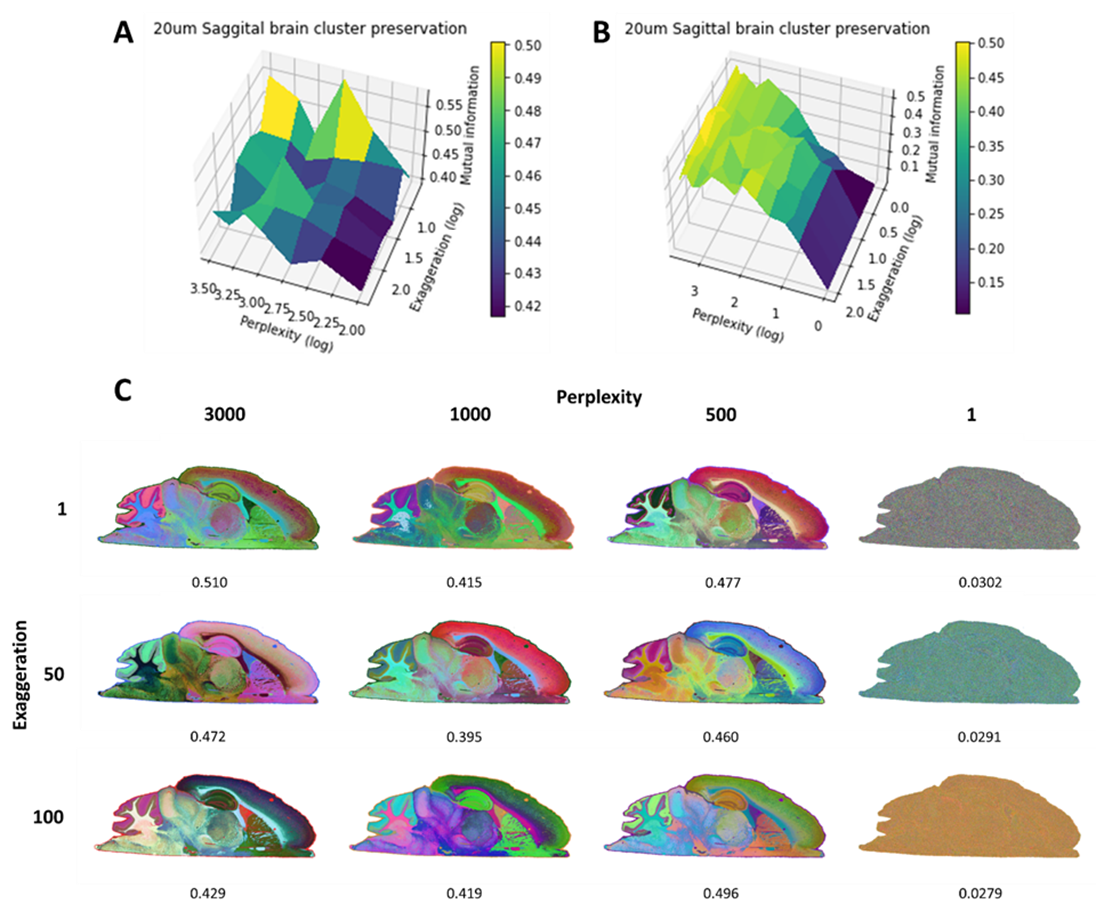


Figure 19 (A) Course grid plot of the t-SNE hyper parameters perplexity vs exaggeration, and their associated cophenetic distance matrix mutual information when compared with the high dimensional cophenetic distance matrix. (B) Finer grid plot of the t-SNE hyper parameters perplexity vs exaggeration, and their associated cophenetic distance matrix mutual information when compared with the high dimensional cophenetic distance matrix, using perplexity values. (C) 3D t-SNE embeddings of the 20 μm sagittal brain dataset, perplexity values along the top, exaggeration along the left.


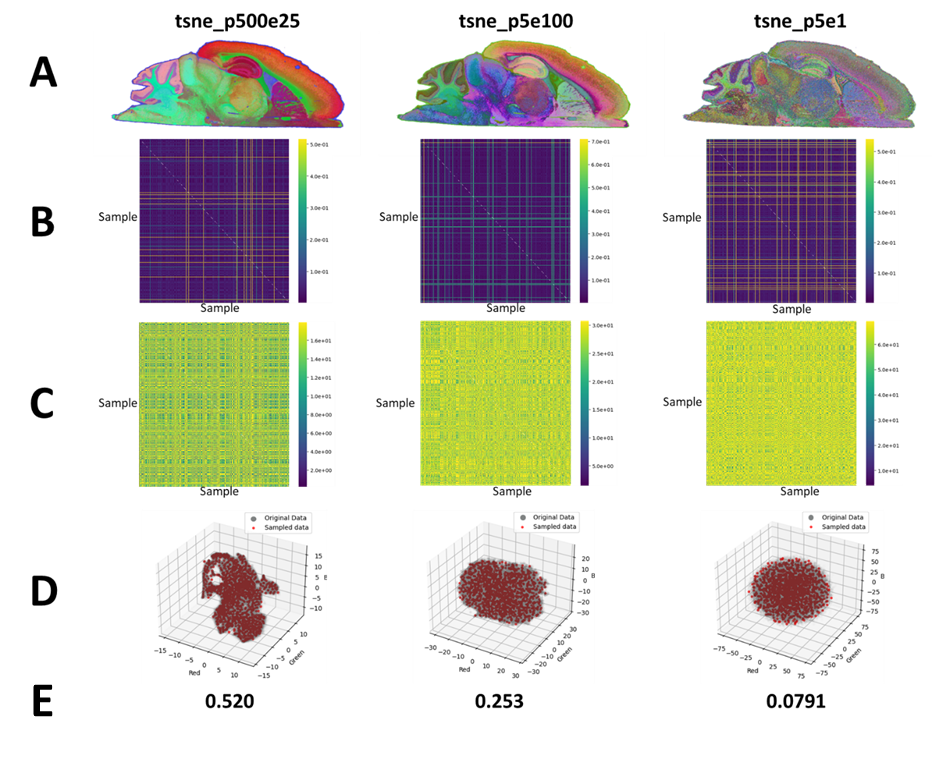


Figure 20 (A) Embeddings for different combinations of t-SNE hyper-parameters. (B) The pairwise cophenetic distance matrix between sampled points for the high dimensional space. (C) The pairwise cophenetic distance matrix between sampled points for the low dimensional space. (D) A 3D scatter plot of the t-SNE embeddings, including labelling of sampled data points. (E) The mutual information between the high dimensional pairwise cophenetic distance matrix, and the t-SNE embedding pairwise cophenetic distance matrix.

Figure 20 shows the embeddings for a range of mutual information scores for this dataset. Even without a reference image to compare these embeddings to, it is clear that *perplexity 5, exaggeration 1* has not performed optimally. Visually, the embeddings look noisy without clear distinction between every group. This is also reflected in the 3D scatter in panel D where the data is very diffuse without clear structure which the mutual information score of ~0.08 reflects; this means that local and global structure has not been preserved well. The embeddings of *perplexity 500 exaggeration 25 and perplexity 5 exaggeration 100* look visually similar in differentiating anatomical regions of the brain, with a difference being that the cerebral cortex of *perplexity 500 exaggeration 25* appearing less noisy than that of *perplexity 5 exaggeration 100*. The cophenetic distance matrix of *perplexity 500 exaggeration 25* in panel C has a much larger proportion of “middle distance” or “mid-near” cophenetic distances compared to the other embeddings in this Figure. And this key difference may be the reason for the greater overall mutual information score. The higher perplexity value allows the embedding process to consider a larger neighbourhood and helps in preserving relationships between “mid-near” points.


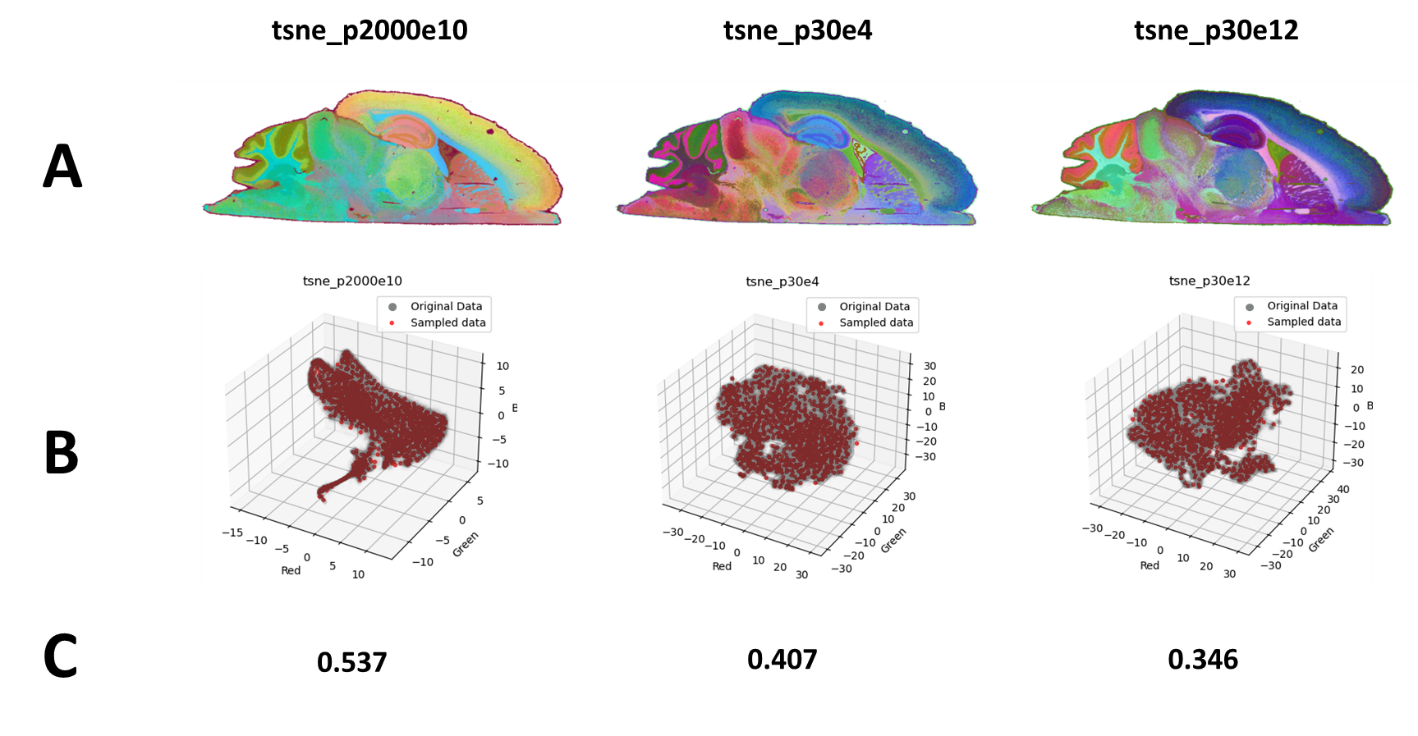


Figure 21 (A) Greatest embedding found by DCI, van der Maaten recommended hyper parameters, and Sklearn default hyper parameters. (B) 3D scatter plots for each embedding. (C) Mutual information scores between high and low dimensional space cophenetic distance matrices.

The visual similarity in anatomical region separation between each embedding in Figure 21 emphasises the need for an objective measure of structure and pattern preservation of dimensionality reduction. Visual inspection of embeddings only, does not provide enough information for an evaluation of local and/ or global preservation of data. A main difference that can be seen in the 3D scatter plots of panel C is that *p2000e10* has a distribution of data which appears to have a more global separation between groups, with a higher perplexity value causing this. The influence of global features dominating this dataset may be the reason that this embedding has the greatest mutual information even though visually, it looks as though local features are not as clearly separated which is due to the high perplexity. Although visual separation of local features may appear poor, overall cluster preservation for this embedding was the most optimum of all produced embeddings.

**Transverse brain dataset**


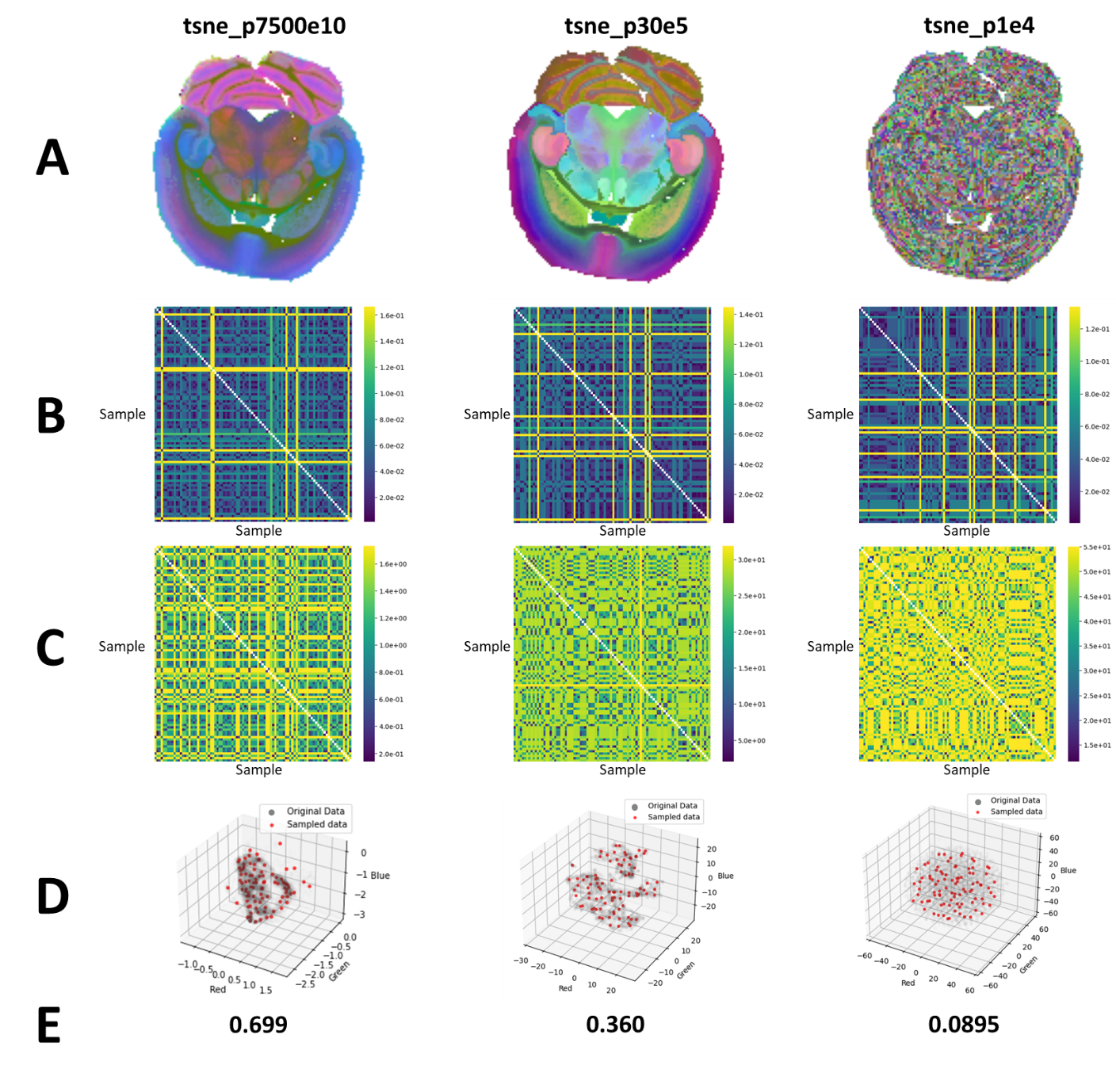


Figure 22 (A) Embeddings for different combinations of t-SNE hyper-parameters. (B) The pairwise cophenetic distance matrix between sampled points for the high dimensional space. (C) The pairwise cophenetic distance matrix between sampled points for the low dimensional space. (D) A 3D scatter plot of the t-SNE embeddings, including labelling of sampled data points. (E) The mutual information between the high dimensional pairwise cophenetic distance matrix, and the t-SNE embedding pairwise cophenetic distance matrix.

Figure 22 demonstrates the embeddings produced across a range of mutual information scores. This ranges from one of the greatest mutual information scores (left) to the worst mutual information score (right). Inspection of the cophenetic distance matrices between high and low dimensional space for *perplexity 7500 exaggeration 10* shows that generally, the relative cophenetic distances between sampled data points has been preserved well – this is with exception to few samples which have a much more distant relationship to other samples in high dimensional space, which do not maintain that relationship in low dimensional space. Other than these samples, all other sampled datapoints appear to maintain their relative order of cophenetic distances well between both dimensions, meaning that local and global cluster relationships have been maintained. The embedding of *perplexity 30 exaggeration 5* doesn’t appear to have preserved the relative cophenetic distances between high and low dimensional space as well as *perplexity 7500 exaggeration 10* when inspecting the matrices in panel B and C. The sampled data points appear more closely related in low dimensional space than they are in high dimensional space, with fewer sampled datapoints which are more globally related; that is, having a greater relative distance to all other samples. As with other dataset results, the *perplexity 1* embedding representing the worst-case embedding is incredibly noisy, without any discernible structure.


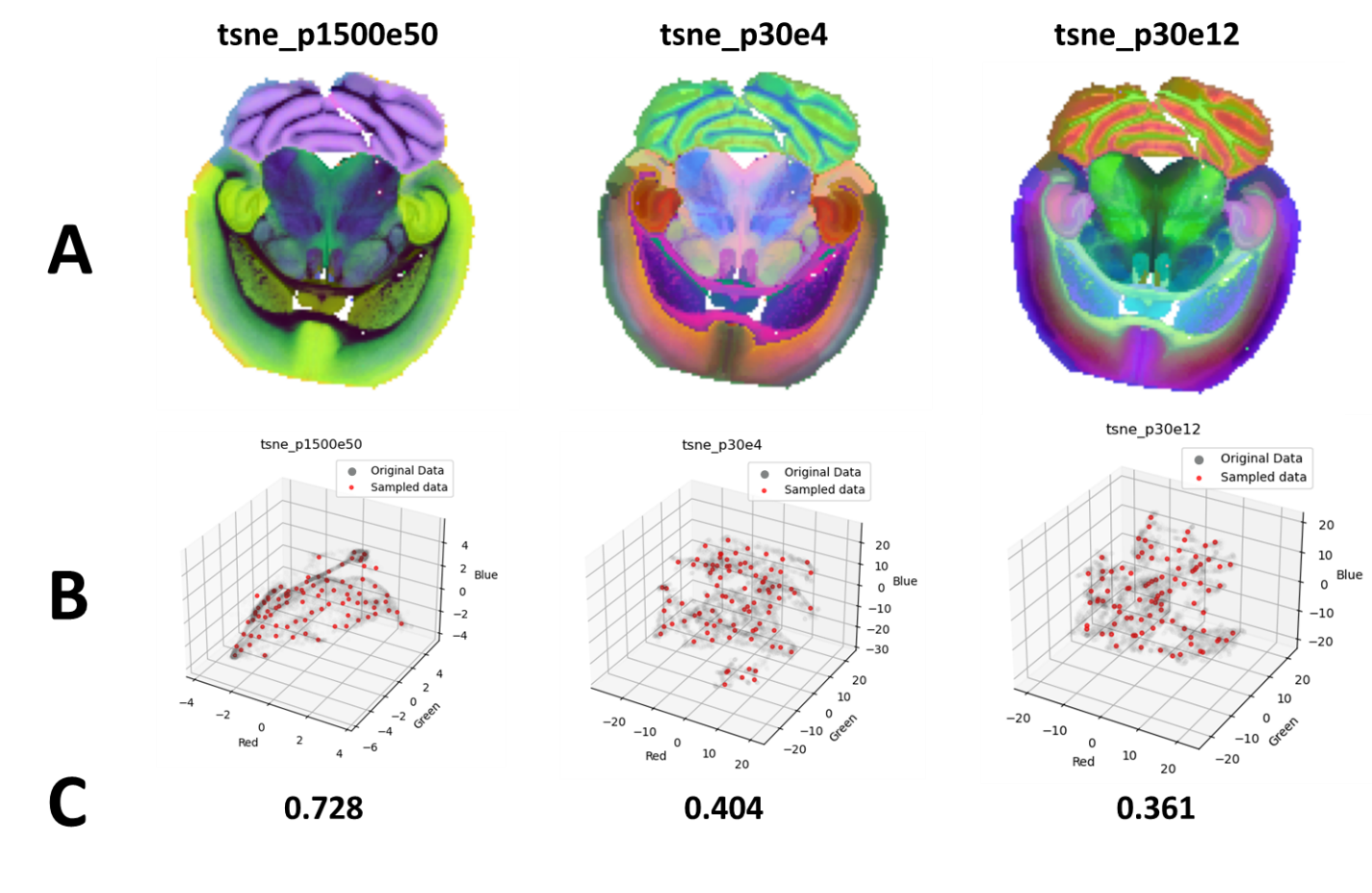


Figure 23 (A) Greatest embedding found by DCI, van der Maaten recommended hyper parameters, and Sklearn default hyper parameters. (B) 3D scatter plots for each embedding. (C) Mutual information scores between high and low dimensional space.


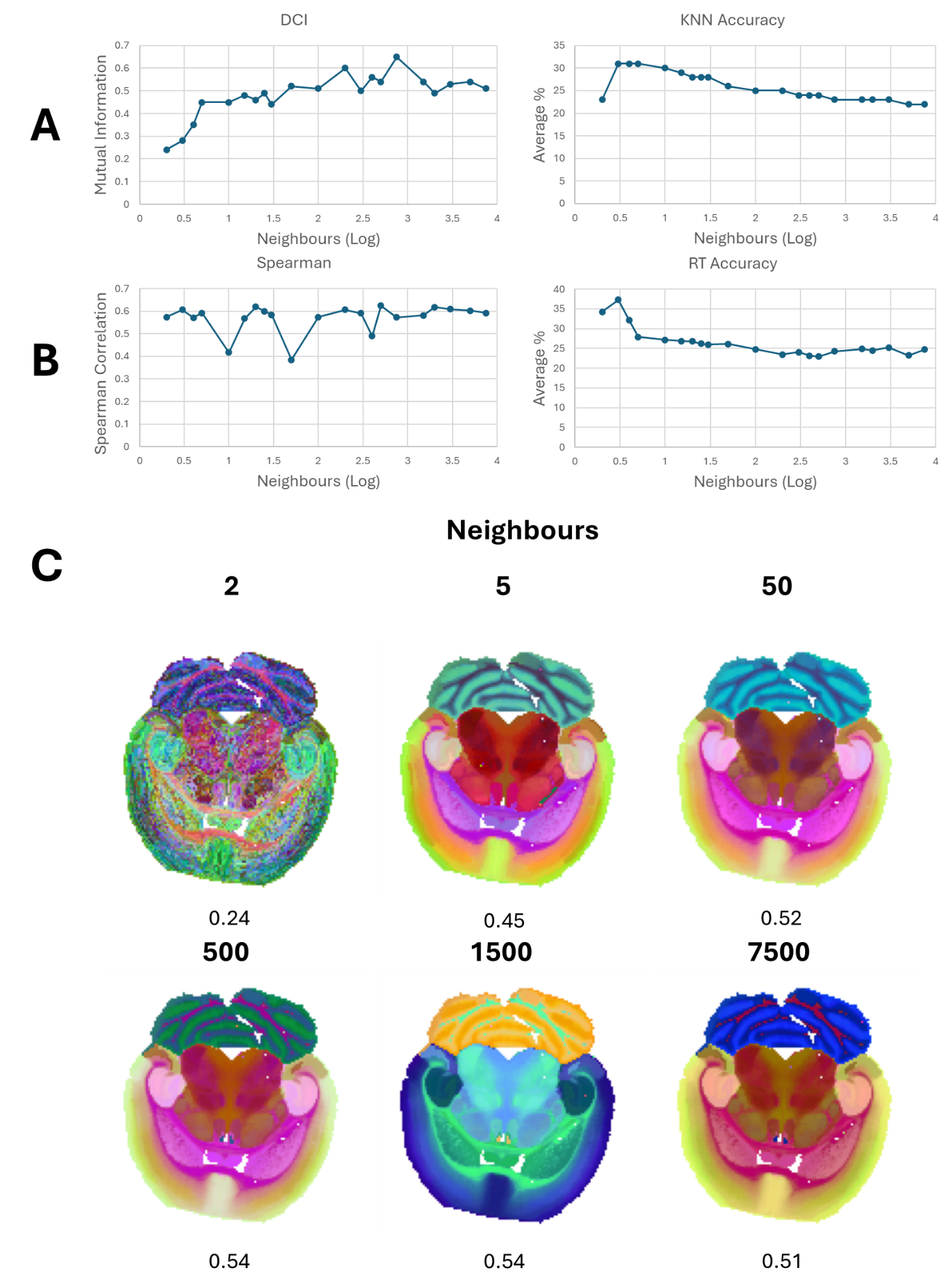


Figure 24 (A) Plots of DCI and KNN accuracy for a range of neighbours, respectively. (B) Plots of Spearman rank and random triplet accuracy for a range of neighbours, respectively. (C) UMAP 3D reduced representation embeddings of a range of neighbours with their associated DCI results.

(1) Venna, J.; Kaski, S. Local Multidimensional Scaling. *Neural Networks* 2006, *19* (6–7), 889–899. https://doi.org/10.1016/j.neunet.2006.05.014.

(2) Griparis, A.; Faur, D.; et al. A Dimensionality Reduction Approach for the Visualization of the Cluster Space: A Trustworthiness Evaluation. In *2016 IEEE International Geoscience and Remote Sensing Symposium (IGARSS)*; IEEE, 2016; pp 2917–2920. https://doi.org/10.1109/IGARSS.2016.7729753.

(3) Siegel, S. Nonparametric Statistics. *Am Stat* 1957, *11* (3), 13–19. https://doi.org/10.1080/00031305.1957.10501091.

(4) Gracia, A.; González, S.; et al. A Methodology to Compare Dimensionality Reduction Algorithms in Terms of Loss of Quality. *Inf Sci (N Y)* 2014, *270*, 1–27. https://doi.org/10.1016/j.ins.2014.02.068.

(5) Zhang, Y.; Shang, Q.; et al. PyDRMetrics - A Python Toolkit for Dimensionality Reduction Quality Assessment. *Heliyon* 2021, *7* (2), e06199. https://doi.org/10.1016/j.heliyon.2021.e06199.

(6) Huang, H.; Wang, Y.; et al. Towards a Comprehensive Evaluation of Dimension Reduction Methods for Transcriptomic Data Visualization. *Commun Biol* 2022, *5* (1), 719. https://doi.org/10.1038/s42003-022-03628-x.

(7) Wang, Y.; Huang, H.; et al. Understanding How Dimension Reduction Tools Work: An Empirical Approach to Deciphering t-SNE, UMAP, TriMAP, and PaCMAP for Data Visualization. *Journal of Machine Learning Research* 2021, *22* (201), 1–73. https://doi.org/arXiv.2012.04456.

(8) Kobak, D.; Berens, P. The Art of Using T-SNE for Single-Cell Transcriptomics. *Nat Commun* 2019, *10* (1), 5416. https://doi.org/10.1038/s41467-019-13056-x.

(9) Dunn, J. C. A Fuzzy Relative of the ISODATA Process and Its Use in Detecting Compact Well-Separated Clusters. *Journal of Cybernetics* 1973, *3* (3), 32–57. https://doi.org/10.1080/01969727308546046.

(10) Lee, J. A.; Verleysen, M. Quality Assessment of Dimensionality Reduction: Rank-Based Criteria. *Neurocomputing* 2009, *72* (7–9), 1431–1443. https://doi.org/10.1016/j.neucom.2008.12.017.

(11) Xu, G.; Gan, S.; et al. Application of Clustering Strategy for Automatic Segmentation of Tissue Regions in Mass Spectrometry Imaging. *Rapid Communications in Mass Spectrometry* 2024, *38* (8). https://doi.org/10.1002/rcm.9717.

(12) Sarretto, T.; Gardner, W.; et al. A Machine Learning-Driven Comparison of Ion Images Obtained by MALDI and MALDI-2 Mass Spectrometry Imaging. *J Am Soc Mass Spectrom* 2024, *35* (3), 466–475. https://doi.org/10.1021/jasms.3c00357.

(13) Smets, T.; Verbeeck, N.; et al. Evaluation of Distance Metrics and Spatial Autocorrelation in Uniform Manifold Approximation and Projection Applied to Mass Spectrometry Imaging Data. *Anal Chem* 2019, *91* (9), 5706–5714. https://doi.org/10.1021/acs.analchem.8b05827.

(14) Abdelmoula, W. M.; Balluff, B.; et al. Data-Driven Identification of Prognostic Tumor Subpopulations Using Spatially Mapped t-SNE of Mass Spectrometry Imaging Data. *Proceedings of the National Academy of Sciences* 2016, *113* (43), 12244–12249. https://doi.org/10.1073/pnas.1510227113.

(15) Tu, A.; Said, N.; et al. Spatially Resolved Metabolomic Characterization of Muscle Invasive Bladder Cancer by Mass Spectrometry Imaging. *Metabolomics* 2021, *17* (8), 70. https://doi.org/10.1007/s11306-021-01819-x.

(16) Linderman, G. C.; Rachh, M.; et al. Fast Interpolation-Based t-SNE for Improved Visualization of Single-Cell RNA-Seq Data. *Nat Methods* 2019, *16* (3), 243–245. https://doi.org/10.1038/s41592-018-0308-4.

(17) Fabian Pedregosa; Gaël Varoquaux; et al. Scikit-Learn: Machine Learning in Python. *Journal of Machine Learning Research* 2011, *12*, 2825–2830.

(18) Virtanen, P.; Gommers, R.; et al. SciPy 1.0: Fundamental Algorithms for Scientific Computing in Python. *Nat Methods* 2020, *17* (3), 261–272. https://doi.org/10.1038/s41592-019-0686-2.
